# Small-Molecule Inhibitors of Amyloid Beta: Insights from Molecular Dynamics—Part B: Natural Compounds

**DOI:** 10.3390/ph18101457

**Published:** 2025-09-28

**Authors:** Mariyana Atanasova

**Affiliations:** 1 Faculty of Pharmacy, Medical University of Sofia, 1000 Sofia, Bulgaria; matanasova@pharmfac.mu-sofia.bg; 2 Centre of Excellence in Informatics and Information and Communication Technologies, 1113 Sofia, Bulgaria

**Keywords:** amyloid beta (Aβ), amyloidosis, oligomerization, fibrils, amyloid beta inhibitors, molecular dynamics (MD, Alzheimer’s disease (AD), natural compounds

## Abstract

Alzheimer’s disease (AD) is the most common form of dementia, characterized by progressive memory loss and cognitive decline. Its key pathological hallmarks include extracellular amyloid plaques composed of amyloid beta (Aβ) peptides and intracellular neurofibrillary tangles formed by hyperphosphorylated tau protein. Although numerous studies have investigated the complex pathology of AD, its underlying mechanisms remain incompletely understood. The amyloid cascade hypothesis continues to be the leading model of AD pathogenesis. It suggests that Aβ aggregation is the initial trigger of neurotoxicity, setting off a cascade of pathological events including inflammation, oxidative stress, tau hyperphosphorylation, synaptic dysfunction, and, ultimately, dementia. Molecular dynamics (MD) is a powerful tool in structure-based drug design (SBDD). By simulating biomolecular motions at the atomic level, MD provides unique insights into molecular properties, functions, and inhibition mechanisms—insights often inaccessible through other experimental or computational techniques. When integrated with experimental data, MD further deepens our understanding of molecular interactions and biological processes. Natural compounds, known for their pleiotropic pharmacological activities, favorable safety profiles, and general tolerability (despite occasional side effects), are increasingly explored for their potential in both the treatment and prevention of various diseases, including AD. In this review, we summarize current findings from MD simulations of natural compounds with anti-amyloidogenic potential. This work builds upon our previous publication, which focused on endogenous compounds and repurposed drugs. The review is structured as follows: an overview of the amyloid cascade hypothesis; a discussion of Aβ oligomeric structures and their stabilizing interactions; a section on molecular dynamics, including its challenges and future directions; and a comprehensive analysis of the inhibitory mechanisms of natural compounds, categorized by their shared structural features.

## 1. Introduction

Alzheimer’s disease (AD) is the most common form of dementia, characterized by memory impairment and cognitive decline. It is a leading cause of death, especially among the elderly [1,2]. The disease is defined by the accumulation of amyloid β (Aβ) plaques, tau protein tangles, oxidative stress, inflammation, neurotransmitter imbalances, and neuronal degeneration, primarily affecting the cortex and hippocampus [3,4]. AD is also classified as an amyloidosis disorder, a group of diseases associated with the abnormal deposition of fibrillar proteins [5]. Current treatments, including acetylcholinesterase inhibitors and NMDA receptor antagonists, alleviate symptoms but do not prevent disease progression [3,6,7,8]. Ongoing research focuses on alternative therapeutic approaches targeting Aβ and tau pathology, though the precise mechanisms underlying AD remain unclear [9,10,11,12,13].

**Figure 1:**
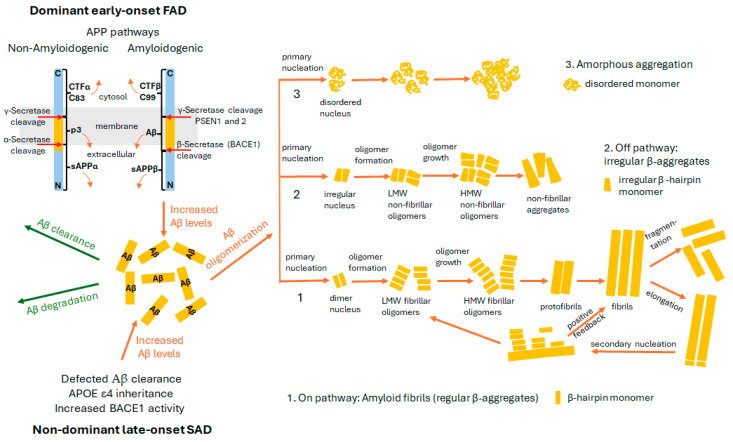
Overview of factors contributing to Aβ accumulation and aggregation pathways in AD. The left panel illustrates the key factors contributing to elevated Aβ levels in both familial and sporadic forms of Alzheimer’s disease (AD), reinforcing the amyloid cascade hypothesis. These include mutations in the APP gene, mutations in PSEN1 and PSEN2, which encode the catalytic subunits of γ-secretase (presenilins 1 and 2), inheritance of the APOE ε4 allele, increased β-secretase 1 (BACE1) activity, and impaired Aβ clearance. The schematic illustrates APP cleavage pathways: in the non-amyloidogenic pathway, APP is cleaved by α-secretase, followed by γ-secretase, producing soluble APPα (sAPPα), a non-toxic peptide fragment p3 (17–40/42 amino acids), and the C-terminal fragment α (CTFα or C83). In contrast, the amyloidogenic pathway involves initial cleavage by β-secretase, yielding sAPPβ, followed by γ-secretase cleavage, which generates the Aβ peptide (1-40/42 amino acids) and C-terminal fragment β (CTFβ or C99). Excess Aβ can undergo clearance, degradation, or self-aggregation through several distinct pathways: (1) fibrillar aggregation pathway: U-shaped β-strand (β-hairpin) monomers aggregate via primary nucleation to form fibrillar oligomers and protofibrils; (2) off-pathway aggregation: irregular β-sheet monomers form disordered oligomers and nuclei, leading to the formation of non-toxic, irregular aggregates; and (3) amorphous aggregation: random coil, disordered monomers associate into non-structured tangles or amorphous aggregates. Secondary mechanisms that further influence Aβ aggregation include fibril elongation, fibril fragmentation (monomer-independent), and secondary nucleation (monomer-dependent), all of which are modulated by fibril concentration. With permission of Atanasova [23], copyright 2025 Pharmaceuticals.

The amyloid cascade hypothesis is the dominant model explaining AD pathogenesis, suggesting that Aβ peptide is the primary driver of neurotoxicity and a cascade of pathological events in the central nervous system (CNS) [9,14]. Aβ aggregation into oligomers and deposits leads to vascular damage, inflammation, oxidative stress, disrupted ionic homeostasis, tau phosphorylation, neurofibrillary tangle (NFT) formation, synaptic loss, cognitive impairment, and dementia [9,14]. Moreover, phosphorylated tau exacerbates Aβ toxicity through a feedback loop [15]. Genetic evidence supports this hypothesis, with APP (amyloid precursor protein), PSEN1, and PSEN2 (encoding two catalytic subunits of γ -secretase) mutations linked to early-onset familial AD (FAD) [16,17], while late-onset sporadic AD (SAD) is associated with the APOE ε4 (apolipoprotein E ε4) allele, impaired Aβ clearance, and increased BACE1 (β—secretase 1, β-site amyloid precursor protein cleaving enzyme 1) [18,19,20,21,22] (Figure 1).

The Aβ peptide is generated through the cleavage of APP, specifically the APP695 isoform found in neurons [24]. In the amyloidogenic pathway, β-secretase and γ-secretase process APP695, resulting in the formation of soluble APPβ (sAPPβ), C-terminal fragment β (CTFβ or C99), Aβ, and APP intracellular domain (AICD) (Figure 1) [20,25,26]. The Aβ molecule is a 4 kDa polypeptide composed of 38–53 amino acids, with Aβ_1-42_ being the most neurotoxic [27,28,29]. It undergoes self-aggregation through a primary nucleation mechanism, forming oligomers, protofibrils, and fibrils. Three aggregation pathways have been proposed based on Aβ_1-42_’s initial secondary structure (Figure 1) [30,31]. In the toxic amyloid pathway, monomers transition from an α-helix or random coil to a β-sheet structure, forming nuclei during the lag phase [32]. Soluble oligomers and protofibrils develop in the growth phase, followed by amyloid fibril formation in the stationary phase [33]. Secondary aggregation mechanisms include elongation and secondary nucleation (monomer-dependent) or fragmentation (monomer-independent) (Figure 1) [34,35,36]. Secondary nucleation accelerates oligomer production once a critical fibril concentration is reached, driving toxic oligomer accumulation [34]. The positive feedback mechanism accelerates the exponential accumulation of toxic oligomers [37,38,39].

Recent studies suggest that disease severity is closely linked to soluble Aβ oligomer levels, highlighting them as key neurotoxic agents [33,40,41,42,43]. These oligomers disrupt cellular functions by interacting with membrane receptors, altering signaling pathways, and forming pore-like structures that compromise neuronal membrane integrity (reviewed in [33]). Consequently, they contribute to calcium imbalance, mitochondrial dysfunction, oxidative stress, ATP depletion, and tau phosphorylation, ultimately leading to synaptic dysfunction and neuronal loss. This shift in understanding has led to the evolution of the amyloid cascade hypothesis into the “oligomer hypothesis” [44].

Current AD treatment strategies primarily target three key approaches: (i) reducing Aβ production using α-secretase activators and β- and γ-secretase inhibitors; (ii) promoting Aβ clearance through immunization, receptor-mediated mechanisms, and modulation of neuroinflammation; and (iii) preventing Aβ oligomerization using amyloid inhibitors, such as small molecules, nanomaterials, nanoparticles, and metal chelators [12,45,46,47,48,49]. A significant advancement in passive immunization led to the FDA approval of the monoclonal antibodies Aducanumab (2021) and Lecanemab (2023) [50,51]. Furthermore, researchers continue to investigate Aβ aggregation promoters to better understand fibrillogenesis and develop potential modulators [52,53,54,55,56,57,58].

## 2. Oligomeric Structures and Stabilizing Interactions

**Figure 2:**
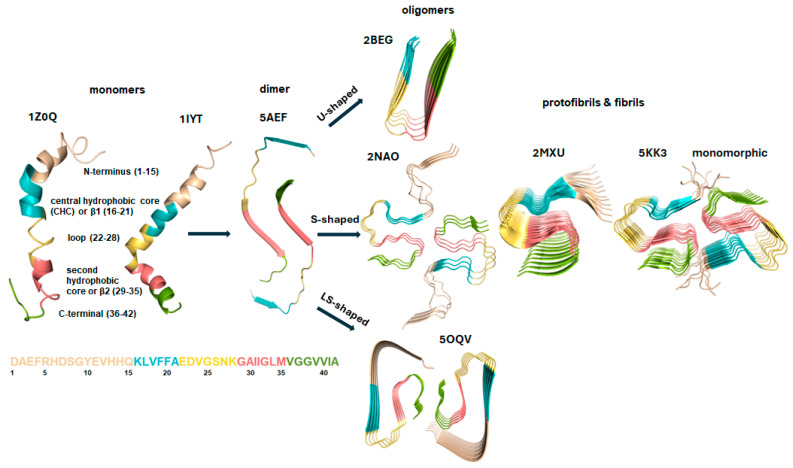
Structural features and aggregation of human Aβ_1-42_. This figure presents the primary and secondary structures of the human Aβ_1-42_ peptide, along with selected aggregate structures retrieved from the Protein Data Bank (www.rcsb.org). Each structure is labeled with its corresponding PDB code. The peptide chain is color-coded according to key regions in its primary amino acid sequence, with every fifth residue numbered: N-terminal hydrophilic/metal-binding region (D1–Q15): beige, central hydrophobic core (CHC) or β1 region (K16–A21): cyan; central hydrophilic loop (E22–K28): yellow; second hydrophobic core (β2) (G29–M35): salmon; and C-terminal segment (V36–A42): green. These structural regions play critical roles in Aβ aggregation. In particular, the C-terminal region is highly hydrophobic and is believed to drive rapid self-assembly through hydrophobic interactions—contributing significantly to oligomer formation [71]. With permission of Atanasova [23], copyright 2025 Pharmaceuticals.

Soluble Aβ oligomers, ranging from 10 to 100 kDa, display significant heterogeneity and dynamic properties. According to the protein folding funnel model, unfolded monomers reside in a high-energy state at the top, while folded intermediates, oligomers, and fibrils descend into lower-energy states [59,60,61,62,63,64]. Amyloid fibrils represent the global free energy minimum, with amyloid crystals occupying the absolute lowest energy state [62,63]. However, the mechanisms governing the conversion from monomers to fibrils remain unclear due to the metastability and variability of oligomers. Factors such as temperature, concentration, and monomer homogeneity play a crucial role in fibrillogenesis. Structural studies using NMR and cryo-electron microscopy have identified various Aβ oligomer conformations, including U-shaped, S-shaped, and LS-shaped structures, some of which have been derived from human samples, as illustrated in Figure 2 [65,66,67,68,69,70].

Amyloid fibrils primarily exhibit a cross-β-sheet structure, with the Aβ backbone aligned perpendicular to the fibril axis [72,73,74,75,76]. In on-pathway oligomers, β-sheet structures are commonly present, whereas Aβ monomers in solution generally remain unfolded (Figure 2). The Aβ_1-42_ sequence can be categorized into five distinct regions: the N-terminal hydrophilic/metal-binding region (D1–Q15), the central hydrophobic core (K16–A21), the central hydrophilic loop (E22–K28), the second hydrophobic core (G29–M35), and the C-terminal segment (V36–A42) (Figure 2) [48].

Studies have shown that intrastrand hydrogen bonds, particularly between I31 and V36, play a crucial role in β-hairpin monomer formation. These bonds break during oligomerization, leading to the formation of interstrand hydrogen bonds that drive aggregation and establish a cross-β-sheet structure [77,78]. The hydrophobic interaction between F19 and L34 stabilizes the β-sheet structure in mature fibrils [79,80,81], while the salt bridge between D23 and K28 further reinforces the turn in the cross-β-structure [82,83,84]. Oligomerization likely involves parallel stacking along the fibril axis, and fibril growth is driven by dimeric Aβ_1-42_ units in a “ying-yang” arrangement [68,69,70]. Key interactions stabilizing quaternary fibril structures include side-chain interactions between M35 and residues such as I31, I32, and G37, G39, and V29 [85,86,87,88,89]. Different Aβ fibril structures are stabilized by distinct interactions. The U-shaped Aβ_17-42_ relies on K28-D23 salt bridges, while the S-shaped Aβ_11-42_ and LS-shaped Aβ_1-42_ are stabilized by K28-A42 salt bridges [67,69,90]. In the LS-shaped form, E11-H6/H13 hydrogen bonds reinforce fibril stability. Additionally, a hydrophobic core involving F4, L34, and V36, along with the K28-A42 salt bridge, plays a key role in maintaining the LS-shaped fibril structure [69].

## 3. Molecular Dynamics Simulations

Understanding how small molecules inhibit oligomerization and designing new inhibitors is challenging due to the vast conformational space of Aβ monomers and oligomers, their metastable nature during fibrillization, and dynamic structural changes. Computational methods, like molecular dynamics (MD) simulations, serve as valuable tools to complement traditional experimental approaches in this research.

MD is a crucial structure-based drug design (SBDD) technique that aids in understanding the properties, functions, and mechanisms of biomolecules. It is the only method capable of simulating biomolecular motions at the atomic level, providing insights not attainable through conventional experiments [91]. By integrating experimental data with MD simulations, a deeper understanding of molecular processes is achieved. In classical, also denoted as conventional, MD (cMD), atoms are modeled as particles with defined positions and velocities, interacting through force fields (FFs) that calculate the potential energy of a system. Specialized force fields are required for different molecular systems, such as proteins (Amber ff14SB [92], GROMOS versions 54A7 and 54B7 [93], CHARMM [94], and OPLS3 [95]), as well as FFs tailored for small organic molecules. For intrinsically disordered proteins (IDPs) like Aβ peptides, specific FFs have been developed to better capture their dynamic behavior [96,97,98,99,100,101]. These include ff99IDPs, ff14IDPSFF [102], ff14IDPs [103], ff03 CMAP [104], ff14SB [92], CHARMM36m [105], andCHARMM36IDPSFF [106]. The suitability of force fields for IDPs depends on various factors, including system design, simulation conditions, sampling methods, and water models. Additionally, force field comparisons are based on diverse criteria such as chemical shifts, secondary structure content, global reaction coordinates, J-coupling, and dissociation constants. For instance, in replica exchange molecular dynamics (REMD) simulations of monomeric Aβ_1-42_, OPLS with the TIP3P water model, AMBER99SBILDN, and CHARMM22 outperformed AMBER99SBILDN-NMR, with the latter three using the TIP4P-Ew water model [107]. In another study comparing force fields for monomeric Aβ_42_, ff14SB and CHARMM36m were recommended over ff99SB, FF14SB_IDPs, and CHARMM36, all of which utilized the TIP3P water model in conventional MD and REMD simulations [108]. As a result, multiple studies report conflicting findings on the most accurate force field for modeling IDPs and protein aggregation. Additionally, various software packages provide specific recommendations for force fields and water models tailored to IDPs. For example, the latest version of Amber24 suggests using the ff99SD force field alongside the general-purpose four-point OPC water model [109,110]. However, newer force fields consistently exhibit greater accuracy than older ones, reflecting ongoing advancements in their refinement.

Classical molecular dynamics (cMD) enables precise calculations by iteratively determining atomic forces in femtosecond time steps, based on Newton’s equations of motion derived from potential energy functions. To address these limitations, advanced sampling techniques have been developed to extend simulation timescales and enhance sampling efficiency [111,112,113,114,115,116,117], as extensively reviewed by Zuckerman [115]. For further details on cMD and advanced MD methods, see references [112,115,116,117,118,119,120,121,122,123].

By utilizing non-Boltzmann factors to maintain a uniform potential energy distribution, the system can overcome energy barriers and explore a wider conformational space. This principle underlies the generalized-ensemble algorithm for enhanced sampling [124,125,126]. A brief overview of enhanced sampling methods in molecular dynamics (MD) is presented in Table 1.

Generalized-ensemble methods (e.g., simulated tempering ST, replica exchange, RE) extend conformational sampling by varying system temperature, while coarse-graining reduces system complexity to access longer timescales. Biasing potential methods (e.g., conformational flooding, metadynamics, umbrella sampling) promote exploration of specific conformations, and accelerated MD techniques modify the potential energy landscape to overcome kinetic barriers. Together, these strategies address the timescale and sampling limitations of conventional MD.

While many advanced techniques effectively address cMD’s timescale limitations, no single method is universally ideal [152,153]. Continuous improvements in MD sampling methods, force fields, and computational algorithms, along with increasing computational power, have significantly extended simulation timescales (some examples can be found [154,155,156]). For instance, Anton 3, a supercomputer designed for MD simulations, can perform over 100 microseconds per day for million-atom systems [157]. High-performance supercomputers like LUMI and others enable cellular-scale simulations [155,158]. Additionally, distributed computing projects like Folding@home have leveraged global computational resources, achieving exascale computing and simulating 0.1 s of the SARS-CoV-2 proteome during the COVID-19 pandemic [159,160,161]. These advancements make MD simulations increasingly indispensable for studying biological systems.

Natural compounds are regarded as safer, with relatively lower cytotoxicity, and offer significant potential as treatments for diseases linked to protein misfolding and aggregation. Although many have been tested in clinical trials, none have yet been approved as an amyloid β inhibitor [162,163].

More than a decade ago, early studies on amyloid β inhibitors using SBDD (molecular docking and MD simulations) methods for fragments and full-length peptides were reviewed, emphasizing limitations such as timescales, implicit solvent models, and scoring function constraints [164]. Since then, only a few reviews have addressed the applications of these methods for Aβ inhibitors, providing limited examples [48,165,166,167]. However, no comprehensive review has focused on MD simulations of natural compounds over the past twelve years, which is the objective of this study. This work follows our previous investigation into endogenous compounds and repurposed drugs [23]. Its goal is to expand the understanding of Aβ inhibitors derived from MD simulations and provide insights that could assist in the design of future in silico and/or experimental studies focused on uncovering the mechanisms behind Aβ inhibition, as well as in the development of novel Aβ inhibitors. The reviewed articles mainly focus on the Aβ_42_ form, including its fragments, monomers, dimers, oligomers, protofibrils, and fibrils. However, simulations on Aβ_40_ were also included in cases where studies on Aβ_42_ were limited, to provide additional insights into a specific inhibitor, when an alternative MD method was used, or when the fibril structure was considerably larger.

The following sections examine small-molecule Aβ inhibitors of natural origin, grouped into three categories based on structural similarity: (1) Scaffold 1, comprising two rings—at least one of which is a phenyl ring—connected by linkers of varying length and type; (2) flavonoid-based scaffolds; and (3) condensed-ring scaffolds. A separate section is dedicated to individual compounds that do not fall into these categories.

## 4. Natural Compounds with Scaffold 1 Inhibiting Aβ

**Figure 3:**
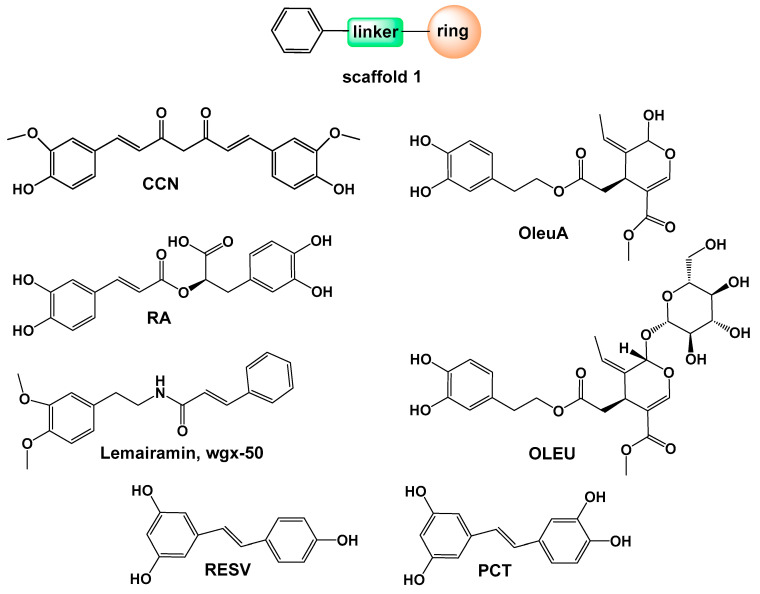
Natural compound Aβ inhibitors with scaffold 1, examined through MD simulations, are shown.

A common structural pattern, referred to as scaffold 1 in Figure 3, is present in this group of natural compounds that inhibit Aβ. It consists of two rings, with at least one being phenyl, connected by linkers of varying lengths and types. This scaffold is found in curcumin (CCN), rosmarinic acid (RA), lemairamin (wgx-50), resveratrol (RESV), piceatannol (PCT), oleuropein (OLEU), and oleuropein aglycone (OleuA) (Figure 3).

### 4.1. Curcumin (CCN)

Curcumin is a key compound found in the rhizomes of Curcuma longa Linn (turmeric), a plant from the Zingiberaceae family [168]. It is a polyphenolic curcuminoid, consisting of two phenolic rings linked by a β-diketone group (Figure 3). CCN has been extensively studied due to its wide range of biological activities, including anti-inflammatory, antioxidant, anti-arthritic, hypolipidemic, cardioprotective, hepatoprotective, anti-diabetic (type II), anti-cancer, and more [168,169,170,171,172,173]. Its lipophilic nature allows it to cross the blood–brain barrier (BBB) [174,175]. The compound’s multifunctional properties are also observed in the treatment of neurodegenerative diseases, such as Alzheimer’s disease (AD), Parkinson’s disease, prion diseases, multiple sclerosis, Huntington’s disease, amyotrophic lateral sclerosis, as well as in managing conditions like anxiety and depression [168,172,176,177].

CCN has been shown in vitro and in vivo to counteract key Alzheimer’s disease mechanisms, including Aβ and tau aggregation and oxidative stress [175,176,178]. It inhibits Aβ aggregation (IC_50_ = 0.8 µM), prevents fibril formation, reduces oligomer toxicity, and destabilizes preformed fibrils by decreasing β-sheet content [175,179,180]. CCN also inhibits tau fibrillization and disassembles existing tau filaments [181].

Curcumin binds to fibrillar tau filaments and Aβ plaques in vivo, promoting amyloid degradation and reducing plaque burden [175,182]. It selectively lowers soluble tau dimers and oligomers, and it can co-crystallize with the β-sheet core of tau [178,183]. The unique structure of CCN, featuring hydrogen-donating phenolic groups and an electron-donating methylene group in the β-diketone linker, supports its antioxidant activity by scavenging reactive oxygen and nitrogen species (RONS), which contribute to oxidative stress and neuroinflammation [184,185,186,187,188].

CCN also reduces the activation of NF-κB (nuclear factor kappa B), a key regulator of pro-inflammatory and pro-oxidant gene expression, and inhibits ROS-generating enzymes such as lipoxygenase, cyclooxygenase, and xanthine oxidase [185,189]. As a natural fluorophore, it distinguishes AD deposits with high sensitivity and specificity, making it a potential imaging agent for early AD diagnosis [172,190]. Additionally, CCN and related curcuminoids inhibit BACE-1, as demonstrated in *Drosophila* models [191].

However, the main challenge in using CCN as a therapeutic agent is its poor pharmacokinetic profile, characterized by low bioavailability due to poor water solubility, low absorption, rapid metabolism, and quick elimination [192]. To address these issues, several strategies have been explored, including chemical and microbial modifications, complexation with phospholipids, and entrapment in liposomes, nanoparticles, or exosomes (reviewed in [193,194]). A novel lipidated CCN formulation called LONGVIDA^®^ has been developed to enhance bioavailability [195,196]. As of February 2023, LONGVIDA^®^ completed a pharmacokinetic study in clinical trials, though the results have yet to be published [197].

CCN is among the most extensively studied compounds in MD simulations due to its diverse roles in diagnosing, preventing, and treating Aβ plaques. Extensive simulations have explored its anti-amyloid mechanisms across different Aβ forms, from monomers to fibrils (Table 2). Ono et al. showed in an in vitro study using fluorescence spectroscopic analysis and electron microscopic studies that CCN dose dependently inhibits Aβ_1-40_ and Aβ_1-42_ fibril formation from the corresponding monomers [179]. Yang et al. reported that CCN inhibits Aβ_1-40_ oligomer formation in vitro, as shown by ELISA, aggregation assays, and EM analysis [175,198]. Even at low physiologically relevant concentrations, CCN suppressed fibril formation, with stronger inhibition at higher doses. In a “seedless” monomer assay (Aβ_1-40_ and Aβ_1-42_ dissolved in HFIP), CCN reduced oligomerization in a dose-dependent manner and blocked Aβ_1-42_ oligomer toxicity in SH-SY5Y cells at 0.1–1 µM. These findings suggest that CCN interacts preferentially with aggregated rather than monomeric Aβ. To further explore this mechanism, MD simulations were performed to model primary nucleation (Aβ_1-42_ monomers) and oligomer formation (Aβ_1-42_ dimers) in the presence of CCN. The first MD study examining CCN’s effect on the Aβ_1-42_ was carried out by Zhao and colleagues in 2012 using an Aβ_1-42_ generated dimer for docking of both CCN tautomers, followed by 500 ns cMD simulations on 20 top-scoring complexes (10 for each tautomeric form—β-diketone and keto-enol) (Table 2) [199]. They found that CCN disrupted Aβ dimer’s β-sheets structure, reducing β-sheet content by 21CCN’s phenyl rings formed π–π stacking with F19 in CHC of Aβ, while dynamic interactions were observed with L34 in the second hydrophobic region and E22 in the loop region. L34 emerged as a central hub in CCN’s movement around the peptide. These findings are consistent with an in vitro study by Banerjee (2014) on Aβ_1-42_ monomers, which showed that CCN reduces the β-sheet structure of Aβ in a time-dependent manner, as revealed by Circular Dichroism (CD) spectroscopy [180]. Additionally, Atomic Force Microscopy (AFM) demonstrated that CCN inhibits β-sheet formation in the presence of Cu^2+^ and Zn^2+^ ions, with a stronger effect on Cu^2+^ due to its higher binding affinity. The study also showed that CCN suppresses Cu^2+^- and Zn^2+^—induced oligomerization and protofibril formation of Aβ_1-42_ monomers. In 2015, a study investigated three main CCN binding sites on the Aβ_1-42_ monomer, identified from molecular docking and MD simulations (Table 2) [200]. The study also examined CCN’s effect in the presence of Cu^2+^ ions, simulating two systems with different Cu^2+^ positions near H13. [180].

Overall, based on nine simulations, CCN was found to reduce Aβ aggregation by stabilizing α-helices, disrupting β-sheets, and chelating Cu^2+^ ions. The results are consistent with the experimental study by Banerjee (2014) [180].

Salamanova et al. (2021) studied CCN’s effect on Aβ_1-42_ monomer folding and structure (Table 2) [201]. They found that CCN stabilized the monomer by increasing α-helix content and reducing β-turns, potentially preventing further aggregation. CCN formed several hydrogen bonds, particularly long-lived ones (>30 ns) with F4, H13, and E11, in the N-terminal hydrophilic region and short-lived ones (>10 ns) with G33 and L24 in the second hydrophobic region. CCN interacted hydrophobically with residues across all Aβ regions, including E3, F4, R5, H6, D7, Y10, and H13 in the hydrophilic region; K16, L17, and F20 in the CHC; M35 in the β2; and V36, V40, and I41 in the C-terminal region. Key interactions included π–π contacts with F4, Y10, H13, H14, and F20, and cation–π interactions with R5 and K16.

Doytchinova and colleagues performed a cMD study on the primary nucleation of 12 full-length Aβ_1-42_ peptides in the presence of either 12 or 36 CCN molecules (Table 2) [202]. Their results showed that CCN interacts with the amyloid ensemble through hydrophobic interactions, π–π stacking with side chains of F4, Y10, F19, and F20, and cation–π interactions with R5, K16, and K28, along with numerous hydrogen bonds. CCN reduced both inter-peptide contacts and hydrogen bonding, while also increasing peptide disorder and reducing flexibility by intercalating into the monomeric core.

Replica exchange solute tempering (REST) was used to simulate one, two, and three unfolded Aβ_1-40_ monomers in a 1:1 molar ratio with CCN (Table 2) [203]. In REST, the solute temperature is varied while the solvent temperature remains constant. Compared to replica exchange molecular dynamics (REMD), REST offers a four- to fivefold reduction in the number of replicas needed, while preserving both the temperature range and sampling efficiency [215]. The study found that CCN reduced β-strand propensity and promoted the formation of random coils and helices. CCN is primarily bound to the CHC via van der Waals interactions.

In vitro investigation using fluorescence spectroscopic analysis and electron microscopic studies on CCN effect on elongation and destabilization of Aβ_1-40_ and Aβ_1-42_ fibrils performed by Ono et al. indicated that CCN dose-dependently inhibits extension and destabilizes preformed fibrils [179]. An in vitro AFM study, performed by Banerjee, reveals that CCN inhibits the spontaneous peptide fibrillization in the presence of Cu and Zn ions [180]. Yang et al. demonstrated in an in vitro Aβ fibrillization model that increasing doses of CCN induced disaggregation of Aβ_1-40_ preaggregated for three days (IC_50_ = 1 µm) [175]. EM analysis further confirmed that CCN inhibited fibril formation in a dose-dependent manner. In vivo studies in Aβ-infused rats and Tg2576 mice showed that CCN exerted potent anti-amyloidogenic activity both before and after extensive amyloid accumulation [175,198]. In vivo studies by Garcia-Alloza et al. in aged APPswe/PS1dE9 mice showed that 7-day CCN treatment prevented the formation of new Aβ deposits, reduced existing deposits by ~30%, and decreased plaque size, indicating a strong disaggregating effect [216]. Moreover, CCN induced a limited but significant reversal of structural changes in dystrophic dendrites, suggesting its ability to counteract existing amyloid pathology and associated neurotoxicity in an AD mouse model. Taken together, these findings highlight CCN’s efficacy across experimental settings. To elucidate the molecular basis of this dual in vitro and in vivo activity, several MD studies were subsequently conducted to investigate how CCN interacts with Aβ at different aggregation states. The interaction mechanism between CCN and a U-shaped protofibril composed of five Aβ_17-42_ chains was explored using a 150 ns cMD simulation (Table 2) [204]. The simulation revealed that CCN primarily binds to the C-terminal residues, with a preference for chain E. It formed π–π stacking interactions with F19 (CHC) and hydrogen bonds with E22 (loop region), M35 (β2 region), and G37 (C-terminal). CCN preferentially destabilized peripheral chains rather than central ones and significantly disrupted D23–K28 salt bridges across all chains. This disruption induced twisting and bending of the peripheral chains, ultimately compromising the structural integrity of the protofibril. These results align with solid-state NMR findings showing that CCN disrupts Aβ_1-42_ fibrils by inducing structural changes in the D23–K28 salt bridge region and near the C-terminus [217].

Bajda and Filipek conducted a similar study of CCN, using it as a reference, by docking it onto a U-shaped pentameric protofibril (Table 2) [205]. Their cMD results showed a significant reduction in the β-sheet structure of the two outermost chains, with the fifth chain becoming completely disordered. Moreover, the number of intermolecular backbone hydrogen bonds was notably decreased.

In a study by Muscat et al., fifty-seven natural compounds were screened via molecular docking against the S-shaped Aβ_11-42_ fibril, followed by MM-GBSA calculations based on short 1 ns MD simulations of the resulting complexes [206]. Among the top candidates, CCN exhibited one of the lowest binding energies and was selected for extended 150 ns production MD simulations (Table 2). The results showed that CCN disrupts the ordered fibril structure by inserting into a pocket within the S-shaped Aβ fibril. This interaction destabilized the fibril, reducing both β-sheet content and inter-chain interaction surfaces.

In a study on CCN derivatives, CCN was used as a reference [207]. Twenty-one docked poses of its keto form and twenty-four of its keto-enol form on an S-shaped hexameric protofibril were subjected to cMD simulations (Table 2). The results showed that CCN partially disrupted the fibril structure, breaking down all β-sheets within the ^12^ VHHQKLVFF ^20^ residue region of the outermost peptide, except for a single remaining β-bridge.

Wang et al. investigated the inhibition mechanism of CCN on Aβ_17-36_ using a coarse-grained model (CGM) and discontinuous molecular dynamics (DMD) simulations (Table 2) [208]. They explored CCN’s effects on monomer binding, oligomerization, disruption of preformed protofilaments, and inhibition of protofilament growth. In the monomer–CCN system (10 molecules), CCN primarily interacted with hydrophobic residues L17, F19, F20, A21, I31, I32, L34, and M35. In a system of eight aggregating monomers and 30 CCN molecules, fibrillization was inhibited, forming disordered complexes. In the third setup, CCN bound to preformed protofilaments but did not dissolve them. Finally, in a system simulating protofilament elongation (protofilament + 8 monomers + 30 CCN), CCN was found to slow the growth process.

In 2018, Tavanti and colleagues employed REMD to identify the binding sites (BSs) of five natural biophenols, including CCN, on Aβ_1-40_ fibrils (Table 2) [209]. They discovered that CCN primarily bound to the β-2 BS, corresponding to the β-sheet region spanning parts of the second hydrophobic and C-terminal segments of Aβ (residues ^31^ IIGLMVG ^37^). This interaction exhibited the strongest binding affinity. Other sites—β-1 (central hydrophobicβ-sheet, ^16^ KLVFFAEDV ^24^), Elbow (loop between β-1 and β-2, ^22^ EDVGSN ^27^), Over (top of the protofibril), and C-terminal (end of the β-2 β-sheet near the cleft entry)—showed moderate to strong affinities. Within the β-2 BS, it primarily interacted with residues M35, G33, and I31. Notably, ligand migration from the Over site to the β-2 site near M35 indicated binding-induced perturbation.

Recently, Khurshid and colleagues conducted a cMD study at an elevated temperature (360 K) to accelerate dynamics and better understand CCN–Aβ interactions (Table 2) [210]. They explored three scenarios with varying CCN concentrations: (1) a single Aβ_1-42_ monomer, (2) 24 Aβ_1-42_ monomers, and (3) 25-mer Aβ_17-42_ fibril. In the first case (1:1 ratio), CCN exhibited non-specific, dynamic interactions with hydrophobic residues (F, L, V, A, I), without forming stable binding. At a 4:1 ratio, CCN disrupted β-sheet structure and increased random coil content. In the second scenario, CCN prevented β-sheet formation, promoting disordered aggregates. At higher concentrations, CCN molecules self-associated before interacting with Aβ, resulting in coarse, disordered structures. In the third scenario, CCN is bound specifically to the Aβ_17-42_ fibril. Molecules either remained isolated or stacked, aligning parallel (dominant) or perpendicular to the fibril axis. In the parallel mode, CCN is intercalated between 2 and 4 β-strands, interacting with Gly33 on different peptides. In the perpendicular mode, CCN is localized near hydrophobic residues (M, I, and V), tracing the ^33^ GLMVG ^37^ motif. Additional molecules entered the hydrophobic core via open fibril ends or bound to the loop between β-strands.

Mladenova et al. evaluated the effects of CCN on Aβ-induced cytotoxicity in SH-SY5Y neuroblastoma cells under simultaneous, pre-, and post-incubation conditions, reflecting immediate protection, prevention of Aβ toxicity, and repair of Aβ-induced damage with restoration of cell viability [218]. Notably, in contrast to previous experimental and in silico findings, CU showed no protective effect; instead, it enhanced Aβ toxicity under all three conditions. A similar effect was reported by Ono et al. in 2012, showing that CCN had minimal interference with Aβ_1-40_ and Aβ_1-42_ oligomerization, with only slight inhibition at a 1:4 peptide/CCN ratio and EC_50_ values of 60.8 and 108.2 µM, respectively [219].

### 4.2. Rosmarinic Acid (RA)

Rosmarinic acid is a natural polyphenol and an ester of caffeic acid, commonly found in plants of the Lamiaceae and Boraginaceae families, including rosemary, sage, basil, oregano, marjoram, and lemon balm [220]. RA exhibits a wide range of biological activities, such as antibacterial, anti-inflammatory, antiviral, antioxidant, anticancer, antiallergic, cardioprotective, antidiabetic, hepatoprotective, nephroprotective, antidepressant, and neuroprotective effects [221,222,223,224,225]. Studies have shown that RA significantly delays disease onset and prolongs lifespan in a mouse model of familial amyotrophic lateral sclerosis [226]. In an AD mouse model, RA alleviated memory impairment caused by Aβ neurotoxicity [227]. Ono et al., using fluorescence spectroscopy and electron microscopy, demonstrated that RA dose-dependently inhibits the fibril formation of both Aβ_1-40_ and Aβ_1-42_ from their monomers [179]. They further showed that RA prevents fibril elongation and destabilizes preformed Aβ_1-40_ and Aβ_1-42_ fibrils in a dose-dependent manner. Furthermore, RA reduces the cellular toxicity and synaptic dysfunction associated with Aβ oligomers, and significantly decreases Aβ deposition in transgenic AD mice [219,228]. RA was found to moderately inhibit Aβ_1-40_ and Aβ_1-42_ oligomerization at a 5:2 peptide/RA ratio and completely inhibit it at 1:4, with EC_50_ values of 25.6 and 10.8 µM, respectively [219]. NMR studies revealed that RA does not bind to monomeric Aβ. Furthermore, RA reduced the cellular toxicity and synaptic dysfunction associated with low-n Aβ_1-42_ oligomers.

RA exhibits low oral bioavailability due to rapid metabolism, which limits systemic exposure and underscores the need for advanced formulations to enhance stability, absorption, and tissue distribution [229]. Owing to its hydrophilic nature, RA shows poor BBB penetration, resulting in limited cerebral bioavailability. Nanoparticle-based delivery systems have been proposed as a potential strategy to overcome this limitation [230,231].

The precise anti-aggregation mechanism of RA remains inadequately understood. A study employing REMD simulations on the Aβ_1-40_ protofibril revealed that RA primarily binds to the N-terminal disordered tails and the β-1 binding site (^16^ KLVFFAEDV ^24^) in CHC (Table 2) [209]. However, the most stable interactions—based on moderate binding free energies—were observed at both the β-1 and β-2 BSs, the latter formed by residues ^31^ IIGLMVG ^37^ from the second hydrophobic region. Key contact residues included K16, V18, and F20 at β-1, and M35, G33, and I31 at β-2. Additionally, RA was found to affect the secondary structure of the double-layer protofibril.

In another study, replica permutation molecular dynamics (RPMD) was employed to investigate the interaction between RA and theAβ_16-22_ peptide (Table 2) [211]. Similarly to REMD, RPMD yields statistically reliable conformational data by initiating simulations from random peptide structures. It utilizes the Suwa-Todo algorithm—a Markov chain Monte Carlo method—to permute temperatures between replicas while minimizing the rejection rate of state transitions [232]. Two systems were analyzed: one with a 1:1 peptide-to-inhibitor ratio and another with a 1:4 ratio. In both cases, RPMD simulations revealed that RA primarily formed hydrogen bonds with E22 and K16. This interaction disrupted the critical electrostatic contact between K16 and E22, thereby inhibiting Aβ oligomerization and preventing the formation and stabilization of the cross-β-sheet fibril structure.

### 4.3. Lemairamin (Gx-50; Wgx-50)

N-[2-(3,4-dimethoxyphenyl)ethyl]-3-phenylacrylamide—also known as lemairamin, gx-50, and wgx-50 (Figure 3)—is a natural compound found in Sichuan pepper (*Zanthoxylum bungeanum*). It has been shown to inhibit Aβ-induced chemotactic migration of microglia and exert anti-inflammatory effects by modulating the TLR4-mediated NF-κB/MAPK signaling cascade [233,234]. Additionally, wgx-50 enhances cognitive function via the GSK-3/CREB pathway [235]. Lemairamin has shown promising anti-Aβ activity, disassembling oligomers, preventing fibril formation, and protecting against Aβ-induced apoptosis in vitro [236]. In transgenic mice, it improved cognitive function, reduced cortical Aβ oligomers by ~60%, and protected neurons. Pharmacokinetic studies confirmed its oral bioavailability and ability to cross the BBB, reaching the brain within minutes and persisting for several hours [236].

The mechanism by which gx-50 destabilizes Aβ protofibrils was explored by Fan et al. using MD simulations (Table 2) [212]. Three potential binding sites were identified: two on the surface of the protofilament, and a third—most energetically favorable and consistently observed in multiple MD replicas—located in the concave region formed by the U-shaped β-strand–loop–β-strand motif. This site lies within the hydrophobic core and interacts with the side chains of I32 and L34. Binding of gx-50 at this site partially disrupted the cross-β-sheet subunit, inducing structural distortion between the first two peptide subunits. This led to increased inter-peptide distances and promoted protofibril destabilization. Supporting evidence included a reduced number of interchain backbone hydrogen bonds, increased hydrophobic contact distances between A21 and V36, and partial disruption of the D23–K28 salt bridge.

### 4.4. Resveratrol (RESV)

Resveratrol (trans-3,4′,5-trihydroxylstilbene) (Figure 3) is a polyphenolic stilbenoid compound found in varying concentrations in red wine and foods such as peanuts, soybeans, pomegranates, mulberries, and grapes. It has been widely reported to possess a broad spectrum of biological and pharmacological properties, including antioxidant, cardioprotective, cancer chemopreventive, anti-inflammatory, antimutagenic, anticarcinogenic, and antiangiogenic effects [237,238,239,240,241,242]. In addition to its diverse therapeutic potential, RESV exhibits neuroprotective effects against Aβ-induced toxicity: it promotes Aβ clearance, reduces plaque formation, and transforms soluble Aβ oligomers, fibrillar intermediates, and fibrils into non-toxic, unstructured aggregates. According to Feng et al., RESV inhibits the cytotoxicity of Aβ oligomers but does not prevent their formation [243]. RESV can bind various Aβ forms—from monomers to fibrils—with a preference for fibrillar aggregates, and it also stains amyloid plaques [244]. Al-Edresi and colleagues reported that RESV fragments Aβ_1-42_ monomers into smaller peptides, suggesting a novel disruption mechanism involving resveratrol-mediated cleavage [245].

RESV absorption is high (around 75%), but its oral bioavailability is very low (<1%) due to rapid and extensive metabolism in the intestine and liver, where it is converted into sulfates and glucuronides [246,247]. RESV needs to be consumed in large amounts to overcome its low bioavailability and rapid conversion into less active metabolites. Strategies to enhance its biological and therapeutic efficacy include chemical modifications (e.g., hydroxylation, amination, and others), combination therapies, and nanoparticle-based encapsulation [248].

Despite extensive experimental evidence supporting RESV’s anti-amyloid activity, its precise molecular mechanism remains unclear. To date, only a limited number of MD simulations have been conducted to investigate RESV’s mode of action (see Table 2).

Coarse-grained modeling (CGM) combined with discontinuous molecular dynamics (DMD) revealed that RESV strongly binds to aromatic residues—particularly F19—in a system consisting of an Aβ_17-36_ monomer and 10 RESV molecules (Table 2) [208]. The key contact residues included L17, F19, F20, A21, I31, I32, L34, and M35. The study demonstrated that RESV inhibits fibril formation, as disordered complexes were observed in simulations involving eight aggregating monomers and 30 RESV molecules. In a separate simulation of a pre-formed protofibril with 30 RESV molecules, RESV disrupted the protofilament structure, leading to the formation of disordered oligomers. Furthermore, in a simulation of protofilament elongation (protofilament plus eight monomers) in the presence of 30 RESV molecules, RESV effectively halted fibril growth.

More recently, Sharma et al. conducted cMD simulations to investigate RESV binding to both the full-length Aβ_1-42_ monomer and a pentameric fibril (Table 2) [213]. In the monomer, RESV formed strong hydrogen bonds with Q15 and D23, while in the pentamer, key interactions involved residues F19, F20, A21, V36, G37, and G38. RESV also engaged in extensive hydrophobic and van der Waals interactions with multiple residues, particularly within hydrophobic cavities and amyloidogenic regions such as V12, H13, K16, F19, F20, D23, V24, and I31. In both Aβ forms, RESV significantly reduced the content of extended β-sheets. Post-simulation analyses and experimental validation demonstrated that RESV prevents monomer aggregation and disrupts the structure of pentameric fibrils, leading to disordered non-toxic aggregates. Additionally, RESV was found to increase the distance between the sulfur atom of M35 and the carbonyl oxygen of I31, contributing to helical destabilization and reducing the likelihood of sulfuranyl radical formation. In summary, RESV interferes with Aβ aggregation, disrupts fibrillar structure, and reduces both α-helix and β-sheet content. Its anti-amyloidogenic activity is primarily mediated through interactions with hydrophobic and structurally critical residues in both monomeric and fibrillar Aβ [213].

### 4.5. Piceatannol (PCT)

Piceatannol, a natural analogue and a metabolite of RESV (Figure 3), is found in the roots of Norway spruces, the seeds of the palm *Aiphanes horrida* (a palm species), and *Gnetum cleistostachyum* [249,250,251]. It also occurs in red wine, grapes, passion fruit, white tea, and Japanese knotweed [252]. PCT exhibits a range of biological activities, including antioxidant, anticancer, and anti-inflammatory effects [252]. Experimental results clearly demonstrated that PCT exerts a broad protective effect against Aβ-induced apoptosis in PC12 cells through a novel PI3K/Akt/Bad signaling pathway, along with downstream mitochondria-mediated and caspase-dependent mechanisms [253]. In Neuro2a cells, PCT was found to induce caspase-3-dependent cell death and autophagy, while reducing γ-secretase activity at higher doses [254]. At lower doses, it significantly increased α-secretase activity, activated MMP-9, and at higher doses, activated cathepsin B. In APPsw-expressing HEK293 cells, PCT markedly reduced Aβ levels, independent of concentration or effects on cell viability [254].

Piceatannol exhibits low solubility and poor bioavailability, undergoing rapid metabolism—particularly glucuronidation—which limits its systemic presence after oral administration [255]. Strategies to address this include chemical modifications, such as prenylation or methylation, and the use of delivery systems like α- and β-cyclodextrins [255]. Additionally, formulation approaches aimed at improving PCT’s solubility and oral bioavailability may further enhance its therapeutic potential [256].

In the previously mentioned study by Muscat et al. (2020), which explored natural compounds targeting the S-shaped Aβ_11-42_ fibril, PCT was subject to cMD simulations (Table 2) [206]. Similarly to CCN, PCT was shown to disrupt the fibril structure by inserting into a pocket formed by the S-shaped Aβ fibril, inducing conformational disorder throughout the structure. It also reduces β-sheet content and decreases inter-chain interaction surfaces. Later (2023), PCT was found to inhibit Aβ peptide aggregation with IC_50_ of 0.48 µM, and also showed activity in DPPH radical scavenging (IC_50_ = 40.4 µM) and acetylcholinesterase inhibition (IC_50_ = 271.74 µM) [257]. In vivo, PCT improved learning behavior in scopolamine-induced ICR mice, while in vitro, it demonstrated neuroprotective effects in SH-SY5Y neuroblastoma cells [257]. Piceatannol was found to protect against Aβ-induced neurite fragmentation in an AD cell model using ndSH-SY5Y cells, with 36% protection efficacy [258]. It also suppressed Aβ-induced neuronal cell death in the same model, showing a 32% inhibitory effect.

### 4.6. Oleuropein (OLEU) and Oleuropein Aglycone (OleuA)

Oleuropein and its aglycone form are natural polyphenols predominantly found in extra virgin olive oil, olive leaves, and drupes (Figure 3) [259,260,261]. These compounds exhibit a broad spectrum of pharmacological properties, including antioxidant, antiviral, anti-inflammatory, antiproliferative, antihyperglycemic, hypocholesterolemic, antimicrobial, and neuroprotective effects [259,262,263,264]. Their potential roles in neurodegenerative diseases such as Alzheimer’s and Parkinson’s have been increasingly recognized, with studies highlighting multiple targets and mechanisms of action—most notably the inhibition of Aβ, tau, and α-synuclein aggregation, as well as the enhancement of Aβ clearance through autophagy induction [260,264,265,266,267,268,269,270,271,272]. In transgenic *C. elegans* models of AD and sIBM, OLE) reduced Aβ plaque formation, decreased toxic oligomers, and improved lifespan and mobility [273]. Protection in CL4176 worms was effective only when OLE was given before Aβ expression. These effects appear dose-dependent and independent of antioxidant activity, indicating that OLE directly interferes with Aβ aggregation. An in vivo study on young to middle-aged TgCRND8 mice fed with OLEU showed improved memory, reduced cortical neuropathology, induced autophagy, and decreased inflammation [270]. Another in vivo study in which Aβ aggregates were injected, with or without OLEU, into the rat brain showed that the Aβ-induced loss of cholinergic neurons (~33%) was prevented when co-aggregated with OLEU [268]. Furthermore, OLEU reduced the formation of toxic Aβ oligomers and attenuated astrocyte and microglial activation, demonstrating its ability to counteract Aβ-induced neurotoxicity and neuroinflammation. These results underscore oleuropein’s potential as a natural neuroprotective agent.

Oleuropein, being a hydrophilic molecule, is sensitive to heat, light, and oxygen, which poses challenges for its stability and pharmaceutical formulation [274]. It exhibits relatively low but notable bioavailability, as most of the compound is rapidly metabolized before reaching the bloodstream in its intact form [274,275]. Various nanocarrier-based delivery systems have been developed to overcome these limitations and enhance oleuropein’s therapeutic potential [274].

In silico studies investigating the anti-amyloidogenic activity of OLEU included 150 ns cMD simulations of OLEU bound to a pentameric, S-shaped Aβ fibril (Table 2) [206]. The simulations revealed that OLEU binds between adjacent peptide chains, leading to significant fibril destabilization. OLEU showed a preference for interacting with residues V18–V24 and N27–I31, resulting in interchain disruption and a marked reduction in both β-sheet content and overall fibril order [206].

The effect of OleuA on preformed Aβ fibrils was also investigated in silico using long-timescale MD simulations (Table 2) [214]. The study began with blind docking to identify potential binding regions, followed by refined docking with the extra-precision (XP) scoring function implemented in the Glide software package (Glide release 2018 (Schrödinger, LLC, New York, NY, 2018). The resulting OleuA–Aβ fibril complex was then subjected to extensive 5 μs MD simulations. The simulations revealed that OleuA induced significant destabilization of the fibril, triggering widespread structural perturbation and ultimately leading to complete disassembly. The compound primarily interacted with residues L17–D23 (the LVFFAED motif), K28, and I31–L34 across all fibril chains. Within the first 500 ns, OleuA disrupted the critical D23–K28 salt bridge between chains A and B. This disruption was accompanied by inter-peptide distances and a marked decrease in inter-chain backbone hydrogen bonds. By the end of the simulation, the peptides had adopted unstructured conformations, with a drastic reduction in β-sheet content. Notably, OleuA exhibited a “cutting” motion throughout the simulation, effectively cleaving and disassembling the highly ordered fibrillar structure [214]. These results contrast with experimental findings by Rigacci et al., who showed that OleuA is most effective when present at the start of Aβ aggregation [269]. It prevents the formation of early toxic oligomers and promotes the generation of stable, harmless protofibrils, structurally distinct from typical Aβ_1-42_ fibrils. When added to preformed fibrils, it does not release toxic oligomers but neutralizes residual toxicity. The study further demonstrated that oleuropein aglycone inhibits Aβ_1-42_ aggregation and cytotoxicity, providing detailed insights into the structural modifications of Aβ_1-42_ in the presence of this polyphenol and their impact on toxicity [269]. Another experimental study by Leri et al., using a combination of biophysical and cell biology techniques to investigate the molecular mechanisms of OleuA on Aβ_1-42_, also contradicts MD findings [271]. The study reported that OleA prevents the formation of toxic Aβ_1-42_ oligomers and inhibits their progression into mature fibrils by interacting with the peptide’s N-terminus. Furthermore, OleA stabilizes oligomers and fibrils, reducing their seeding activity and limiting aggregate–membrane interactions in human neuroblastoma SH-SY5Y cells.

## 5. Natural Compounds with Flavonoid Scaffold Inhibiting Aβ

**Figure 4:**
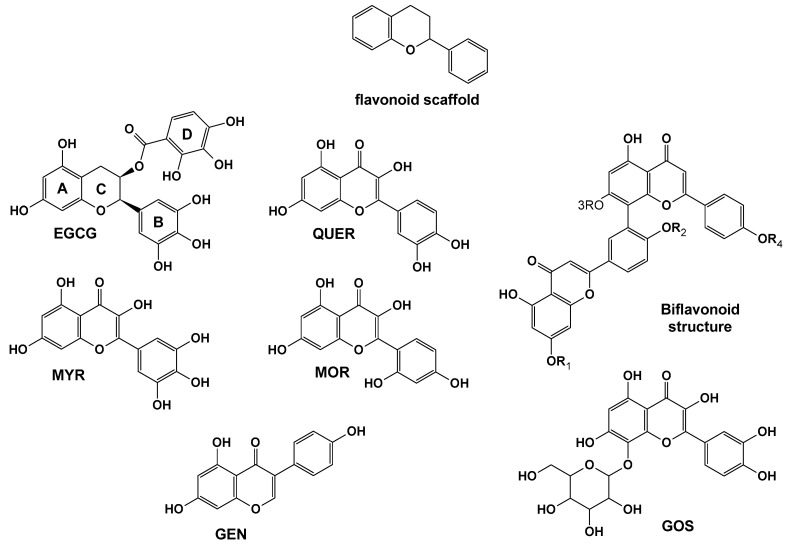
Structures of natural compounds with a flavonoid scaffold that have been analyzed using MD simulations. The substituents for the amentoflavone-type biflavonoids are as follows: AMF―R1 = R2 = R3 = R4 = H; SQF―R1 = CH3, R2 = R3 = R4 = H; BIL―R2 = CH3, R1 = R3 = R4 = H; STF―R3 = CH3, R1 = R2 = R4 = H; PCF―R4 = CH3, R1 = R2 = R3 = H.

The flavonoid scaffold, illustrated in Figure 4, is a common structural motif among natural compounds with demonstrated anti-amyloid activity. Members of this class, whose mechanisms have been explored through MD simulations, include (–)-epigallocatechin-3-gallate (EGCG), myricetin (MYR), quercetin (QUER), morin (MOR), genistein (GEN), gossypin (GOS), and amentoflavone-type bioflavonoids such as amentoflavone (AMF), sequoiaflavone (SQF), bilobetin (BIL), sotetsuflavone (STF), and podocarpusflavone (PCF).

### 5.1. (-)- Epigallocatechin-3-gallate (EGCG)

EGCG is a natural polyphenol and plant-derived flavonoid primarily found in the leaves and stems of the tea plant *Camellia sinensis* (Theaceae family). It is the major active component of green tea and is largely responsible for its potent antioxidant properties, as well as its anti-inflammatory and anticarcinogenic effects [276,277,278,279,280,281,282]. The EGCG molecule consists of two triphenolic groups (Figure 4), which are key to its reactive oxygen species (ROS) scavenging activity [276,277]. It has been shown to inhibit xanthine oxidase and lipid peroxidation [276,279]. EGCG can cross the BBB and reach the brain parenchyma in animal models [283,284]. Numerous studies have demonstrated its anti-amyloidogenic and neuroprotective properties [285,286,287]. It protects against Aβ-induced neuronal apoptosis, and directly binds to mature Aβ and α-synuclein fibrils, converting them into non-toxic, amorphous aggregates [285,288,289,290,291]. EGCG modulates tau hyperphosphorylation, enhances non-amyloidogenic APP secretion through protein kinase C activation, and promotes APP cleavage via α-secretase [288,292,293,294,295]. By shifting APP processing toward the non-amyloidogenic pathway, EGCG increases sAPP-α production and reduces Aβ generation in Swedish mutant APP-expressing neurons. In Tg APPsw mice, EGCG treatment further lowered Aβ levels and plaque burden, supporting its neuroprotective efficacy in vivo [292].

Despite these promising biological effects, EGCG suffers from poor bioavailability due to its physiological instability, which remains a major challenge for its clinical development as a therapeutic agent [283,296]. Strategies to enhance its stability and bioavailability include nanocarrier systems such as bilosomes, prodrug approaches, structural modifications, and co-formulation with other nutrients [297,298,299,300].

Experimental studies show that EGCG effectively inhibits fibrillogenesis by directly binding to natively unfolded Aβ peptides, preventing their conversion into toxic, on-pathway aggregation intermediates [290]. This action redirects amyloid fibril formation toward off-pathway, highly stable oligomers. Instead of producing β-sheet-rich amyloid, EGCG favors the formation of unstructured, nontoxic Aβ oligomers, indicating a broad modulatory effect on protein aggregation pathways in neurodegenerative diseases. EGCG was found to inhibit Aβ aggregation and toxicity; however, heteronuclear solution-state NMR revealed only weak, nonspecific binding, leaving its precise binding mode unresolved [301]. The first cMD simulation investigating the interaction between the Aβ_1-42_ monomer and EGCG provided detailed insights into their binding mechanism through thorough trajectory analyses (Table 3) [302]. The study revealed that EGCG inhibits β-sheet formation in a dose-dependent manner. Key residues involved in the interaction include F4, R5, F19, F20, K28, G29, L34, M35, V36, G37, and I41. Among these, the side chains of F4, F20, M35, and I41, along with the main chains of K28 and G29, contributed predominantly to the nonpolar van der Waals component of the binding free energy. In contrast, the main chains of G29 and G37 contributed significantly to the polar component by forming hydrogen bonds. Additional hydrogen bonds were observed with F20, K28, and G37. Notably, residues such as F20 and I41 engaged in both nonpolar interactions via their side chains and hydrogen bonding through their main chains.

Extensive REMD and quantum chemical calculations were conducted to examine the effect of a single EGCG molecule on six Aβ_16-22_ monomers (Table 3) [303]. The Aβ_16-22_ monomeric fragment was generated using PyMOL v. 1.5.0.4 package [304]. The study revealed that EGCG engages in π–π stacking interactions with F19 and F20 residues (from the CHC) from different peptide units while reducing interchain contacts. The authors suggested that EGCG may accelerate oligomer formation, as the helical intermediates typically observed during oligomerization were absent in the EGCG-containing system but present in the reference system. Although EGCG did not alter the overall β-sheet content compared to the control, it reduced the size of the β-sheet domains—higher-order structures that are potentially associated with increased toxicity.

Zhang and colleagues investigated Aβ dimerization using REMD simulations in the presence and absence of 10 EGCG molecules (Table 3) [305]. They identified key residues interacting with EGCG via their main chains—R5, G29/A30, G37/G38/V39, and A42—as well as those interacting through their side chains, including F4, Y10, F19/F20, I31/I32, M35/V36, V39, and I41. The EGCG molecules formed stable complexes with the dimerizing peptides by embedding themselves at the monomer–monomer interface, primarily interacting with hydrophobic residues. EGCG significantly reduced interchain interactions and induced notable changes in the secondary structure, morphology, and binding free energy of the resulting complex compared to the dimer formed in the absence of the inhibitor.

REST, previously described in Section 4.1 for CCN, was similarly applied to systems containing one, two, and three unfolded monomeric Aβ_1-40_ peptides in a 1:1 molar ratio with EGCG (Table 3) [203]. The simulations revealed that EGCG reduces the propensity of β-strand formation and the likelihood of helical and turn structures. EGCG has been shown to remodel Aβ fibrils into small, spherical oligomers, as evidenced by NMR studies [306,307] and further supported by combined electron microscopy, circular dichroism, and thioflavin T-binding assays [291]. Additionally, EGCG inhibited the formation of hydrophobic contacts between F19 and L34.

**Table 3:** Summarized technical data and key findings from the reviewed MD simulations. While most studies employed cMD, some utilized alternative approaches, which are indicated accordingly. The years indicate the progression of technical developments, computational capacity, and related research findings over the past twelve years.

FF/Water Model	Duration per System, ns	Aβ Length/PDB ID/Type (Monomer/Dimer/ (Proto-) Fibril)	Inhibitor *	Main Findings	Ref.
GROMOS96 53a6/SPC/300 and 330 K	300	Aβ_1-42_/1IYT/monomer	5 and 10 EGCG	inhibits β-sheet dose-dependently; key contact residues: F4, R5, F19, F20, K28, G29, L34, M35, V36, G37, and I41; forms H- bonds with F20, K28, G29, G37, and I41; hydrophobic contacts with F4, F20, K28, G29, M35, and I41	2011 [302]
REMD/Amber99SB-ILDN/TIP3P	NA	Aβ_16-22_/generated/six monomers	EGCG	two ways for preventing Aβ oligomerization—by accelerating oligomerization or by reducing β-sheet content	2017 [303]
REMD/OPLS-AA/SPC	200	Aβ_1-42_/1IYT/dimer	10 EGCG	contacts with main chains of R5, G29/A30, G37/G38/V39, A42, and with side chains of F4, Y10, F19/F20, I31/I32, M35/V36, V39 and I41; forms a stable complex with the dimer and leads to remarkable changes, including secondary structure, morphologies, and interchain interactions	2013 [305]
REST/GROMOS 54a7/SPC	100–800	Aβ_1-40_/2M9R/mono-, di-, and trimer	EGCG in ratio 1:1	reduces β-sheet, increases turn and helical structures; interacts with the CHC of Aβ	2020 [203]
AMBER99SB-ILDN/TIP3P	1000	Aβ_1-42_/5OQV/tetramer, protofibril	20 EGCG	disrupts N-terminal (D1–G9) and C-terminal (K28–A42) segments; increases the kink angle around Y10; decreases H-bonds in the H6–E11 segment, while increasing them in the E11–H13; interrupts the stabilizing K28–A42 salt bridge; interacts with F4, R5, D7, Y10, E11, H13, H14, K28, L34, I41, and A42, via H-bonds, π–π and cation–π interactions; key interactions: H-bonds with E11, π–π stacking with H14 and Y10, with the COO^−^ group of A42 and a cation–π contact with K28	2020 [308]
AMBER99SB-ILDN/TIP3P	300	Aβ_1-42_/5OQV/tetramer, protofibril + POPC/POPG (7:3) membrane	20 EGCG	disrupts protofibril in a lesser extent; reduces structural stability in the D1–G9 and K28–A42 regions, as well as in both hydrophobic cores: F4–L34–V36 (core 1) and L17–F19–I31 (core 2); weakens intrachain salt bridges in the K28–A42 region; distinct inhibition mechanism and binding mode; preferably binds to F4, H6, E11, H13, Q15, L17, F20, and L34; notable contacts: π–π stacking with F4 (from the inner surface) and hydrophobic contacts with L17 and L34; slows the adsorption dynamics by reducing anion–π and cation–π interactions between Y10/H14 and the lipid bilayer; prevents membrane thinning, perturbation and destabilization, induced by protofibril; a dual effect—destabilizing protofibrils and protecting membrane integrity	2021 [309]
CHARMM36m/TIP3P	1000	Aβ_1-42_/5OQV/pentamer, protofibril	3 EGCG	four BSs were identified; residues defining each BS are: F19 for BS1, E3—for BS2, I41—BS3, and E11 and H13 for BS4	2020 [310]
DMD + CGM/PRIME20/implicit solvent effects	50,000	Aβ_17-36_/NA/ (1) monomer (2) 8 monomers (3) pre—formed protofilament (octamer) (4) pre + formed protofilament (octamer) + 8 monomers	(1) 10 EGCG (2) 30 EGCG (3) 30 EGCG (4) 30 EGCG	(1) contacts F19, F20, L34, M35 (2) prevents fibrilization, forming disordered complexes (3) perturbs the protofilament structure, forming a disordered oligomer (4) dissolves the protofilament during its elongation	2017 [208]
REMD/GROMOS 57a7/SPC	50 per replica	Aβ_1-40_/2LMN/decamer, protofibril	EGCG	primary BSs: N-terminal and β-2; secondary BSs: Elbow and β-1; forms interactions with M35, G33, and I31 at β-2, and with K16, V18, and F20 at β-1; disrupts the fibril secondary structure; β-sheets is unfolding of the two outermost monomers; affects the secondary structure of the double-layer protofilament	2018 [209]
GROMOS96 53a6/SPC	100	Aβ_17-42_/2BEG/monomer	(1) 1 MYR (2) 2 MYR (3) 6 MYR (4) 10 MYR	H-bonding is dominant; changes β-sheet content into coil; penetrates Aβ core; forms self-clustered Aβ–MYR complexes at higher molar ratios	2018 [311]
GROMOS 53a6/SPC	100	(1)Aβ_1-42_/2BEG/monomer (2) Aβ_1-42_/2BEG/pentamer, protofibril	10 MYR	(1) forms six H-bonds; transforms β-sheet structure into a random coil; (2) forms five H-bonds; decreases the number of backbone H-bonds between monomers, resulting in loose and uncondensed aggregates; negligible changes in β-sheet structure; converts into expanded, fragile non-toxic amorphous aggregates; the rigidity is destroyed	2020 [30]
RPMD/AMBER parm14SB/TIP3P	100 per replica	Aβ_16-22_/NA/monomer	(1) 1 MYR (2) 4 MYR	in (1) and (2) disrupts the key electrostatic attraction between K16 and E22, as forming H-bonds with E22 in (1), and with E22 and K16 in (2)	2020 [211]
REMD/GROMOS 57a7/SPC	50 per replica	Aβ_1-40_/2LMN/decamer, protofibril	QUER	binds at β-1 BS and at the top of the protofibril (the Over BS); the most stable complexes are at β-2 and β-1 BSs; contacts with M35, G33, and I31 at β-2, and with K16, V18, and F20 at β-1; leads to perturbation of the fibril secondary structure; influences the secondary structure of the double-layer protofilament	2018 [209]
GROMOS96 53a6/SPC	(1) 6 × 150 per system (2) 3 × 250—750 per system until rmsd was stable for 100 ns	(1) Aβ_1-42_/1IYT/monomer (2) three distinct configurations of Aβ_1-42_ dimers—(i) two monomers/1IYT/, (ii) fibril-derived/2BEG/and (iii) soluble oligomer-derived [312]	(1) 2 and 10 MOR (2) 4 MOR	(1) 2 MOR—two BS– (i) at N-terminal, as CHC (L17–A21) contacted C-terminal, Aβ_1-42_ was more compact, tending to collapse; (ii) at F20 of CHC and other hydrophobic residues, elongated Aβ_1-42_; 10 MOR covered Aβ_1-42_, restricting it from collapse and α-helix and β-sheet interconversion; (2) (i) interfacial binding of MOR clusters in the vicinity of hydrophobic residues, directly competed inter-peptide’s interactions; contacts with F19 and F20 from CHC, F4, L34, M35, and V36; β-sheet contend is unchanged; altered quaternary but not secondary structure; *surface* binding to principally polar residues—Y10, H13, E22, D23, and K28, plus F19 and F20, significantly decreases β-strand content; strongly affects secondary structure but dimerization (aggregation) is comparable to control; (ii) fibril-derived dimer structure remains unaffected even though the D23–K28 salt bridge is disrupted; (iii) β-strand converts into random coil, decreases the strength of interaction between peptides	2012 [313]
GROMOS96 53a6/SPC	100	Aβ_17-42_/2BEG/monomer	(1) 1 MOR (2) 2 MOR (3) 6 MOR (4) 10 MOR	H-bonding is dominant; changes β-sheet content into bend; penetrates the core of Aβ, forming self-clusters of Aβ–MOR complexes	2018 [311]
GROMOS 53a6/SPC	100	(1)Aβ_1-42_/2BEG/monomer (2) Aβ_1-42_/2BEG/pentamer, protofibril	10 MOR	(1) forms five H-bonds; transforms β-sheet structure into a random coil; (2) forms four H-bonds; decreases the number of backbone H-bonds between monomers, resulting in loose and uncondensed aggregates; negligible changes in β-sheet structure; converts into expanded, fragile, and nontoxic amorphous aggregates; destroys protofibril rigidity	2020 [30]
CHARMM/TIP3P	2 × 60	Aβ_1-42_/2BEG/pentamer, protofibril	10 GEN	stabilizes Aβ pentamer; reduces the decrease in β-sheet content; contacts L17, F20, E22, I31, G33, M35, and V39; preferably locates at the C-terminal β-sheet groove near G33 and M35	2018 [314]
Amber99-ILDN/TIP3P	150	Aβ_11-42_/2MXU/pentamer, fibril	GOS	inserts into a pocket formed within the S-shaped Aβ fibril; disorders the whole fibril; reduces the β-sheet content and inter-chain interaction surface	2020 [206]
ff99SB/TIP3P	1000 (1 µs)	Aβ_1-42_/2NAO/hexamer (two trimers), fibril	biflavonoids: AMF, SQF, BIL, STF, and PCF	promote disaggregation; preferably bind to the N-terminal pocket of the second trimer; π–π interactions with F4, H6, Y10, H13, and H14, resulting in a significant decrease in β-sheet content due to H-bonding of R2/R3 OH groups of biflavonoids to the peptide backbone	2021 [315]

As most acronyms are identical to those in the previous table, only the new ones are listed below: EGCG—(-)-Epigallocatechin-3-gallate; MYR—myricetin; QUER—quercetin; MOR—morin; GEN—genistein; GOS—gossypin; AMF—amentoflavone; SQF—sequoiaflavone; BIL—bilobetin; STF—sotetsuflavone; PCF—podocarpusflavone, CHC—central hydrophobic core. * number of molecules is specified when different from 1.

This finding aligns with a saturation transfer difference (STD) NMR study showing that EGCG substantially remodels interactions between Aβ monomers and the oligomer surface [316]. The primary binding site was identified as the CHC. Across all three simulated systems, EGCG exhibited a stronger binding affinity than CCN, with van der Waals interactions being the dominant contributing factor.

In vitro studies showed that EGCG inhibits Aβ aggregate formation and protects SH-SY5Y human neuroblastoma cells against Aβ-induced cytotoxicity, restoring cell viability to 77.4% [317]. In vivo studies in a transgenic AD mouse model showed that EGCG, administered either intraperitoneally or orally, reduced brain amyloid plaque burden [292]. It reduces Aβ accumulation and improves cognition in SAMP8 mice, with effects linked to increased expression of neprilysin (NEP), a key Aβ-degrading enzyme [318]. It was demonstrated that EGCG remodels mature Aβ_1-40_ fibrils primarily by binding to hydrophobic sites, blocking thioflavin T interaction, and redirecting fibril structure [319]. Another experimental study, using DEST-based NMR, relaxation, chemical shift projection, fluorescence, dynamic light scattering, and electron microscopy, investigated how EGCG remodels Aβ_1-40_ oligomers [307]. It was found that EGCG binds cooperatively to multiple sites on Aβ oligomers with ∼10-fold higher affinity than monomers, reducing solvent exposure and shifting β-region contacts from direct to tethered, which impairs seeding and interferes with secondary nucleation of toxic assemblies. N-terminal residues shift oppositely, preventing monomer release, while structural changes in both Aβ and EGCG (notably the B and D rings) underlie EGCG’s neuroprotective remodeling mechanism [307]. A recent study by Zhan et al. investigated the inhibitory mechanism of EGCG on the full-length LS-shaped tetrameric Aβ_1-42_ protofibril (Table 3) [308]. The authors initially simulated tri-, tetra-, pentameric protofibrils, as well as nonameric fibril, to identify the most stable fibrillar structure. The tetrameric protofibril was found to be the most stable and thus was selected for detailed analysis of EGCG’s inhibitory effects. EGCG was shown to disrupt key regions of the fibril, particularly the N-terminal segment (D1–G9) and the C-terminal segment (K28–A42), which includes the second hydrophobic core and the terminal region. It increased the kink angle around Y10, contributing to destabilization of the fibril. EGCG also altered the hydrogen bonding network, decreasing the number of H-bonds in the H6–E11 segment while increasing them in the E11–H13 region. Crucially, it disrupted the stabilizing salt bridge between K28 and A42, a hallmark of the LS-shaped fibril conformation. The compound primarily bound to aromatic residues (F4, Y10, H13, H14), charged residues (R5, D7, E11, K28), the deprotonated carboxyl group of A42, and hydrophobic residues (L34, I41) via π–π interactions, hydrogen bonds, and cation–π interactions. EGCG molecules were located on the surface of the LS-shaped protofibril. The key interactions contributing to destabilization included hydrogen bonds with E11 and π–π stacking with H14 and Y10 (which disrupted the H6–E11 H-bond network), and interactions with the COO^−^ group of A42 and a cation–π contact with K28 (which broke the K28–A42 salt bridge).

In a follow-up study, the same group performed multiple cMD simulations of the tetrameric protofibril in the presence and absence of 20 EGCG molecules, in a mixed POPC/POPG (7:3) lipid bilayer environment [309]. They observed that EGCG had a weaker destabilizing effect on the protofibril in the membrane environment. Nevertheless, EGCG still reduced structural stability in the D1–G9 and K28–A42 regions, as well as in both hydrophobic cores: F4–L34–V36 (core 1) and L17–F19–I31 (core 2). EGCG weakened intrachain salt bridges in the K28–A42 region and interactions within both hydrophobic cores. The binding mode of EGCG differed in the membrane environment. The compound showed a preference for binding to F4, H6, E11, H13, Q15, L17, F20, and L34. Notable interactions included T-shaped π–π stacking with F4 (from the inner surface) and hydrophobic contacts with L17 and L34. EGCG also slowed the adsorption dynamics of the protofibril onto the membrane by reducing anion–π and cation–π interactions between Y10/H14 and the lipid bilayer. Additionally, EGCG was found to mitigate membrane thinning and perturbation caused by protofibril binding, thereby enhancing membrane stability. Overall, EGCG exhibits a dual effect—destabilizing Aβ_1-42_ protofibrils while also protecting membrane integrity.

A study by Acharya et al. explored the effect of EGCG on the secondary structure of Aβ_1-42_ by integrating in vitro immune-infrared sensor measurements with in silico approaches, including molecular docking, molecular dynamics (MD) simulations, and ab initio calculations [310]. Molecular docking was first performed against the LS-shaped fibril, identifying three potential EGCG BSs: the first and most favorable involved EGCG inserting into the fibril structure; the second was located near the hydrophilic N-terminus; and the third near the hydrophobic C-terminus. All three EGCG–fibril complexes were subsequently subjected to MD simulations, with technical details summarized in Table 3. During the simulations, all three binding sites remained stable, and a fourth, previously unidentified binding site was discovered. This novel site is located within the fibril, nestled between the two folds of the N-terminal region and the end of the β2 segment at the start of the C-terminal region. It was found to be more frequently occupied than the other three sites. Residue analysis revealed that the fourth site is defined by E11 and H13, while the first, second, and third sites are primarily associated with F19, E3, and I41, respectively.

A CGM combined with DMD simulations was used to investigate the inhibition mechanism of EGCG on amyloid-beta (Table 3) [208]. Four different systems were analyzed. In the first system, comprising an Aβ_17-36_ monomer and 10 EGCG molecules, EGCG formed numerous contacts with the peptide, primarily targeting residues F19, F20, L34, and M35. The second system, containing eight aggregating monomers and 30 EGCG molecules, showed the formation of disordered complexes, suggesting EGCG’s ability to inhibit fibrillization. In the third setup, involving a pre-formed protofilament and 30 EGCG molecules, EGCG disrupted the protofilament, leading to the formation of disordered oligomers. In the fourth simulation, which modeled fibril elongation using pre-formed protofilament and eight additional monomers, the presence of 30 EGCG molecules resulted in protofilament disassembly.

Using REMD, as previously discussed in Section 4.1 and Section 4.2, a study on a decameric Aβ_1-40_ protofibril found that EGCG preferentially binds to the N-terminal region—characterized by disordered tails—and to the β-2 binding site, a β-sheet composed of residues ^31^ IIGLMVG ^37^ encompassing the second hydrophobic region and part of the C-terminus (Table 3) [209]. Two additional secondary binding sites were identified: the Elbow region, which connects the β1 and β2 sheets, and the β1 site, a β-sheet formed by the central hydrophobic region of Aβ, ^16^ KLVFFAEDV ^24^. Calculated binding free energies indicated that EGCG forms stable complexes at both the β2 and β1 sites. At β2, EGCG interacts primarily with M35, G33, and I31, while at β1, it engages with K16, V18, and F20. Notably, the interaction with M35 appears to “chaperone” this residue, inducing a bend that alters the secondary structure of the Elbow region—a structural feature critical for fibril assembly. Furthermore, EGCG disrupts the hydrophobic contact between L34 and F19, which is known to play a key role in fibril initiation, oligomer stability, elongation, and cellular toxicity [80]. During the simulations, unfolding of β-sheets in the two outermost monomers was observed, leading the authors to conclude that EGCG binding disrupts the oligomer’s double-layered structure.

EGCG has been shown to remodel large, mature amyloid-β fibrils into smaller, amorphous aggregates that are nontoxic to mammalian cells [291]. Mechanistic studies indicate that EGCG binds directly to β-sheet-rich aggregates and induces conformational changes without disassembling them into monomers or small diffusible oligomers. The MD simulations not only align closely with experimental observations but also provide complementary mechanistic insights, thereby reinforcing the validity of the experimental findings [290,291,307].

### 5.2. Myricetin (MYR)

Myricetin (also known as cannabiscetin) is a natural phenolic compound from the flavonol class, along with quercetin (QUER) and morin (MOR) (Figure 4). It is found across various plant families, including Myricaceae, Pinaceae, Primulaceae, Polygonaceae, and Anacardiaceae [320,321,322,323,324,325,326]. MYR occurs both in its free form and as O-glycosides in a wide range of edible plants such as berries, vegetables, dock, chard, broad beans, tea, and red wine [327]. Although it has low water solubility, its solubility increases significantly in basic aqueous media due to deprotonation [328,329]. It is most stable at pH 2, with degradation strongly influenced by both pH and temperature [330]. When administered orally, MYR exhibits poor bioavailability due to limited absorption, primarily resulting from its solubility limitations [331]. Strategies such as nanosizing and forming inclusion complexes with compounds like cyclodextrins can markedly enhance its solubility, dissolution rate, and ultimately, its oral bioavailability and therapeutic efficacy [329,332,333]. Despite these pharmacokinetic challenges, MYR displays a broad spectrum of biological activities, including antioxidant, anticancer, antidiabetic, anti-inflammatory, antiviral, antiplatelet, cardioprotective, hepatoprotective, anti-osteoporotic, and neuroprotective effects [320,329,333,334,335]. Notably, many pharmacological studies still employed oral administration of myricetin. In the context of AD, MYR has demonstrated promising effects: it improves learning and memory in streptozotocin-induced rat models, suppresses D-galactose-induced cognitive impairment in mice, inhibits acetylcholinesterase in scopolamine-induced memory impairment, and prevents cognitive decline in similar models [336,337,338]. In vitro studies have shown that myricetin reduces Aβ_1-40_ and Aβ_1-42_ levels by enhancing α-secretase expression and inhibiting β-secretase activity [339]. Ono et al. (2003) investigated the in vitro effects of MYR, QUER, and MOR (discussed in Section 5.3 and Section 5.4) on Aβ_1-40_ and Aβ_1-42_ fibril formation, elongation, and destabilization [179]. All three compounds dose-dependently inhibited fibril formation and extension and promoted the destabilization of preformed fibrils. Their effective concentrations (EC_50_) ranged between 0.1 and 1 µM [340]. In a further study, Ono et al. 2012 found that MYR moderately inhibits Aβ_1-40_ and Aβ_1-42_ oligomerization at a 5:2 peptide/MYR ratio and completely inhibits it at 1:4, with EC_50_ values of 12.4 and 7.0 µM, respectively. In addition, MYR reduced the cellular toxicity and synaptic dysfunction associated with low-n Aβ_1-42_ oligomers [219].

Several studies have investigated the effects of MYR on Aβ aggregation [30,211,311]. A cMD study examining various ratios of myricetin with U-shaped Aβ_17-42_ monomer revealed that MYR intercalates into the Aβ core, forming self-clustered Aβ–MYR complexes at higher molar ratios (see Table 3) [311]. This flavonoid destabilized the peptide structure by converting β-sheet regions into coil conformations. Hydrogen bonding was identified as the primary intermolecular interaction, with myricetin forming contacts with the carbonyl and amine groups of Aβ.

In another study, Gargari et al. conducted MD simulations of MYR with both monomeric and pentameric U-shaped protofibrils (see Table 3) [30]. They found that MYR exerted a dual anti-amyloid mechanism in both systems. In the monomeric form, MYR completely disrupted the β-sheet structure, converting it into random coils. In the protofibril system, MYR transformed the compact structure into loose, uncondensed aggregates by reducing the number of interchain hydrogen bonds. As a result, MYR remodelled rigid, toxic protofibrils into expanded, fragile, and nontoxic amorphous aggregates, effectively redirecting the aggregation pathway. Notably, MYR’s effects on β-sheet structures differed between the two systems: while the β-sheets were entirely disrupted in the monomeric form, they remained partially intact in the protofibrillar form. An early study by Ladiwala et al. (2010) reported that MYR does not block Aβ aggregation but remodels prefibrillar oligomers and fibrils into unstructured, insoluble aggregates [341]. They concluded that MYR promotes nonspecific interactions between Aβ monomers. They further proposed that MYR remodels existing soluble oligomers into disordered aggregates that are unable to progress into fibril formation.

Additionally, RPMD simulations (as discussed in Section 4.2) were conducted on systems containing Aβ_16-22_ peptide and MYR in 1:1 and 1:4 ratios (see Table 3) [211]. In both cases, MYR primarily interacted with E22 through hydrogen bonding, while in the 1:4 system, K16 was also involved. This interaction enabled MYR to disrupt the electrostatic attraction between K16 and E22—a key interaction required for the formation of the antiparallel β-sheet structure in Aβ fibrils. These findings are in good agreement with NMR studies showing that MYR binds to monomeric Aβ at specific residues, including R5, S8, G9, H13, K16, D23, and I31 [219].

### 5.3. Quercetin (QUER)

Quercetin is a widely distributed flavonoid found in various plant parts—such as flowers, bark, stems, roots, leaves, fruits, and vegetables—across more than fifteen plant families, including Moraceae, Apiaceae, Brassicaceae, Moringaceae, Rosaceae, Asparagaceae, and Hypericaceae [342]. Numerous plant-based foods are rich sources of QUER, including rocket, dill, coriander, capers, fennel, broccoli, onions, okra, apples, cherries, berries, elderberries, juniper berries, red grapes, seeds, buckwheat, nuts, green tea, red wine, bee pollen, and olive oil [342,343,344,345]. It exhibits a broad spectrum of biological activities, including anti-inflammatory, antibacterial, antiviral, antifungal, antioxidant, anticancer, antidiabetic, anti-asthmatic, antiallergic, antihypertensive, anti-obesity, neuroprotective, and anti-Alzheimer’s effects. Its anti-Alzheimer’s activity is attributed to several mechanisms, such as the reduction in oxidative stress (both directly and indirectly), suppression of neuroinflammation, inhibition of tau hyperphosphorylation, and both direct and indirect inhibition of Aβ fibrillization [343,344,345]. QUER, along with MYR (see Section 5.2), and morin (MOR, see Section 5.4), has been shown to block Aβ fibril formation and elongation, and to destabilize preformed Aβ fibrils in a dose-dependent manner [340]. Furthermore, in vitro studies have demonstrated that quercetin can reversibly bind to the amyloid fibril structures of both Aβ oligomers and monomers [346].

Quercetin’s hydrophobic nature results in poor water solubility, limiting its absorption and causing precipitation in the digestive tract [347]. It also exhibits low bioavailability and stability, reducing its effective utilization by the body. Various strategies, including nanoparticle encapsulation, complex formation, and co-administration with compounds such as bromelain or fatty acids, are being developed to enhance its solubility, protect it from degradation, and improve absorption, thereby increasing overall bioavailability [348,349,350,351].

Despite extensive biological assays demonstrating quercetin’s broad pharmacological potential and pleiotropic effects, relatively few studies have investigated its mechanisms for inhibiting preformed Aβ fibrils and the Aβ aggregation process. A REMD study identified the binding sites of quercetin on Aβ_1-40_ protofibrils (Table 3) [209]. The study revealed that QUER preferentially binds similarly to the β-1 and Over binding sites. The β-1 site corresponds to the β-sheet formed by the central hydrophobic residues ^16^ KLVFFAEDV ^24^, while the Over site refers to the top region of the protofibril. The results showed that QUER binds moderately to all identified binding sites, with the most stable complexes formed at the β-1 and β-2 sites. Key contact residues included K16, V18, and F20 at β-1, and M35, G33, and I31 at β-2, which represent the junction between protofilaments. Binding at these regions may disrupt the elongation process or affect protofibril stability. Additionally, quercetin was found to alter the secondary structure of terminal monomers and reduce the stability of the oligomer’s double-layered structure.

### 5.4. Morin (MOR)

Morin is a polyphenolic bioflavonoid and an isomer of QUER (Figure 4). It occurs naturally in plant species from the Moraceae, Rosaceae, and Fabaceae families. MOR is a yellow pigment predominantly found in the branches of white mulberry, guava and its leaves, apple skin, onion, almond, Osage orange, as well as in figs, sweet chestnut, jackfruit, red wine, seaweed, tea, coffee, and cereal grains [352,353]. It exhibits a broad spectrum of pharmacological effects, including antioxidant, anti-inflammatory, anticancer, antidiabetic, antimicrobial, anti-arthritic, gastroprotective, nephroprotective, hepatoprotective, cardioprotective, and neuroprotective activities [352]. Moreover, MOR has demonstrated anti-amyloidogenic and fibril-destabilizing effects [340,354].

Morin exhibits low aqueous solubility and poor oral bioavailability, primarily due to rapid degradation, instability in the acidic gastric environment, limited intestinal permeability, and extensive first-pass hepatic metabolism [355]. Strategies to improve its bioavailability and stability include nanodelivery systems such as nanoparticles, niosomes, and self-emulsifying formulations, which enhance solubility and protect the compound from degradation [356,357,358].

Lemkul and Bevan conducted extensive cMD simulations on Aβ_1-40_ and Aβ_1-42_ monomers, as well as on three dimer models: two separate monomers, preformed fibril-derived dimers, and soluble oligomer-derived dimers [313]. In the monomer systems, the molar ratio of MOR to peptide was varied (2:1 and 10:1), while a 2:1 ratio was used in the dimer systems. Although the study provided detailed insights into all systems, for clarity, only the Aβ_1-42_ systems and their corresponding findings are discussed here (see Table 3). At the lower molar ratio (2:1), MOR had minimal impact on the structure of monomeric Aβ_1-42_. However, at the higher ratio (10:1), it significantly altered both the secondary and tertiary structures of the peptide. During dimerization simulations of two monomers, two distinct MOR binding sites were identified: an interfacial binding site, located at the dimerization interface, where MOR influenced the quaternary structure; and a surface binding site, where MOR binding led to a marked decrease in β-sheet content and an increase in helical content. The preformed fibril- and oligomer-derived dimers remained structurally stable, showing only minor perturbations upon MOR binding. Overall, MOR primarily affected the tertiary and quaternary structures of both monomeric and dimeric Aβ_1-42_, leading to conformations distinct from the controls. These altered structures may represent the early formation of “off-pathway” aggregates [313]. These findings are consistent with the experimental results of Ladiwala et al., who investigated the structurally similar MYR molecule [341]. They reported that MYR does not block Aβ aggregation but remodels prefibrillar oligomers and fibrils into insoluble, disordered aggregates incapable of forming fibrils.

Similarly to the effects observed with MYR, a classical MD study investigating various molar ratios of morin with U-shaped Aβ_17-42_ monomers found that MOR intercalated into the peptide’s core, forming self-clusters of Aβ-MOR complexes. This interaction destabilized the U-shaped structure, converting its β-sheet content into bends. The dominant intermolecular forces were hydrogen bonds formed between MOR and the carbonyl and amine groups of Aβ (see Table 3) [311].

Furthermore, in an MD study by Gargari et al., both MOR and MYR were evaluated in monomeric and pentameric Aβ systems, demonstrating that morin, like myricetin, exerts a dual anti-amyloid mechanism (see Table 3) [30]. In the monomeric Aβ system, MOR converted the β-sheet structure into coils. In the pentameric protofibril system, although the β-sheet structure was retained, the rigid “steric-zipper” motif was significantly disrupted, leading to the formation of unstructured, amorphous, and nontoxic aggregates.

### 5.5. Genistein (GEN)

Genistein (Figure 4) is the most abundant isoflavone among phytoestrogens and is widely found in the Leguminosae family, including soybeans, fava beans, lupines, green lentils, alfalfa, and peas. It exhibits a broad spectrum of biological activities, including antioxidant, anti-inflammatory, anticancer, antidiabetic, and anti-Alzheimer’s effects [359,360,361,362]. GEN shown anti-amyloid-β activity by lowering Aβ levels and reducing amyloid plaque deposition in AD mouse models [363,364]. These effects are attributed to genistein’s modulation of peroxisome proliferator-activated receptor γ (PPARγ), which increases apolipoprotein E (ApoE) secretion and promotes Aβ clearance from the brain. Additionally, GEN improves cognitive function, enhancing hippocampal learning, recognition memory, and discrimination ability. GEN inhibits Aβ generation via two primary mechanisms: (i) blocking β-secretase (BACE1) activity by upregulating α-secretase through the protein kinase C (PKC) signaling pathway [365,366,367]; (ii) reducing presenilin levels by downregulating ubiquitin 1 expression (Figure 1) [368]. Genistein has limited bioavailability due to poor aqueous solubility and rapid metabolic degradation [369].

Ren et al. investigated genistein’s anti-amyloid properties through both experimental and computational approaches [314]. Their results showed that GEN effectively inhibits Aβ monomer fibrillization at all tested concentrations, especially at the early stages, by preventing the conformational transition from random coil to β-sheet. In contrast, GEN had minimal effect on the aggregation of preformed Aβ seeds. Complementary MD simulations were performed with a system containing 10 GEN molecules randomly placed around an Aβ pentameric protofibril (Table 3). The simulations aligned with the experimental findings, showing that GEN stabilizes the protofibril and prevents β-sheet loss. Genistein preferentially localizes in the C-terminal β-sheet groove, where it strongly interacts with residues G33 and M35.

### 5.6. Gossypin (GOS)

Gossypin (Figure 4) is a natural flavone found in hibiscus species of the Malvaceae family, including *Hibiscus vitifolius* (tropical rose mallow, also known as Japa or Karupatti), *H. esculentus*, and *H. furcatus* (Panchavam in Malayalam). It has demonstrated a range of pharmacological activities, including anticancer, antioxidant, anti-inflammatory, anti-diabetic, and neuroprotective effects [370,371,372,373]. GOS has been shown to reduce lipid peroxidation in a dose-dependent manner while increasing the levels of superoxide dismutase, catalase, glutathione, and thiols [374]. It is highly water-soluble and hygroscopic but has been associated with some side effects, such as irritation to the skin, eyes, and respiratory tract [375,376].

In the previously mentioned study by Muscat et al., which investigated natural compounds targeting the S-shaped Aβ_11-42_ fibril, GOS was selected for cMD simulations (Table 3) [206]. Similarly to CCN and PCT, GOS was found to disrupt the fibril by inserting into a pocket formed within the S-shaped Aβ fibril, inducing conformational disorder throughout the fibril. It also reduced the β-sheet content and the inter-chain interaction surface. Despite these computational predictions, no experimental data currently confirm gossypin’s anti-amyloidogenic effects.

Given that GOS is a glycosylated form of gossypetin, in vivo studies in 5xFAD AD model mice demonstrated that gossypetin treatment improved spatial learning and memory by significantly reducing both the number and size of Aβ plaques in the hippocampal dentate gyrus and cortex [377]. It also lowered overall brain Aβ levels, thereby ameliorating behavioral deficits. Additionally, gossypetin has been reported to inhibit both Aβ and tau aggregation in vitro [378].

### 5.7. Amentoflavone-Type Biflavonoids

Amentoflavone-type bioflavonoids are natural polyphenols composed of two apigenin units linked by a C–C bond (Figure 4). These compounds are found in various gymnospermous plants, including *Cunninghamia lanceolata* of the cypress family (Cupressaceae), which contains amentoflavone (AMF) and sequoiaflavone (SQF); *Cephalotaxus harringtonia* var. *fastigiata* (Japanese plum yew), which contains bilobetin (BIL); *Torreya nucifera* (Japanese nutmeg-yew), which contains sotetsuflavone (STF), both from the Taxaceae family; and *Podocarpus macrophyllus* var. *macrophyllus* (Japanese yew), which contains podocarpusflavone (PCF) from the Podocarpaceae family [379]. Biflavonoids have shown a range of pharmacological activities, including anti-influenza, anti-inflammatory, antiviral, neuroprotective, and anti-Alzheimer’s effects, notably anti-amyloid-β and anti-BACE1 activities [380,381,382,383,384,385,386,387]. AMF has a stable chemical structure resulting in poor water-solubility and therefore oral bioavailability [388]. Strategies such as amorphous solid dispersions (ASDs) can markedly improve its solubility and bioavailability by enhancing dissolution rates and membrane permeability, thereby increasing its therapeutic efficacy in cancer treatment and other potential applications [389].

AMF and its monomethoxy derivatives—SQF, BIL, and PCF—were found to inhibit Aβ_1-40_ aggregation at ~5 µM [386]. In vitro, AMF showed high-affinity binding to Aβ fibrils (K_i_ = 287 nM), regulated the formation of neurotoxic soluble Aβ_1-42_ oligomers, and selectively labeled amyloid plaques in AD mouse brains [387].

Five amentoflavone-type biflavonoids—AMF, SQF, BIL, STF, and PCF (Figure 4)—which are known to inhibit Aβ fibril formation and disaggregate preformed fibrils [386,387], were further evaluated using thioflavin T fluorescence assays, combined with in silico molecular docking and molecular dynamics simulations (Table 3) [315]. Earlier studies identified AMF as the most potent inhibitor of Aβ fibrillization and the most effective at disrupting preformed fibrils [390]. Atomic force microscopy confirmed that AMF directly disrupted Aβ_1-42_ fibrillar structures, converting β-sheet fibrils into amorphous aggregates [390]. In the present study, thioflavin assays showed that the disaggregating activity of these biflavonoids followed the order: AMF < PCF < SQF < BIL < STF [315]. To explain these differences in silico analyses were performed to provide mechanistic insight into their disaggregation activity. The study employed an S-shaped Aβ fibril model consisting of two dimeric layers with C_2_ symmetry (PDB: 2NAO). Molecular docking revealed that the biflavonoids preferentially bind to a pocket at the N-terminus of the fibril. Analysis of 1 µs MD trajectories showed that these biflavonoids induce conformational changes in the fibril, leading to disaggregation. The compounds formed stabilizing π–π interactions with N-terminal aromatic residues F4, H6, Y10, H13, and H14, contributing to a significant reduction in β-sheet content. A key factor in β-sheet destabilization was the presence of hydroxy substituents at the R2 and R3 positions (Figure 4), which enabled hydrogen bonding with the peptide backbone. This structural feature correlated with the experimental EC_50_ values observed for the five compounds: AMF (0.70 µM) < PCF (1.08 µM) < SQF (1.89 µM) < BIL (2.75 µM) < STF (12.5 µM) [315].

## 6. Natural Compounds with Condensed-Ring Scaffold Inhibiting Aβ

**Figure 5:**
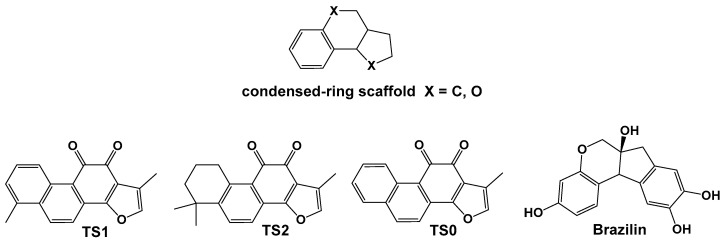
Structures of natural compounds with a three-condensed-ring scaffold investigated using MD simulations.

In this section, we examine natural compounds featuring a three-condensed-ring scaffold, as shown in Figure 5, that exhibit anti-amyloidogenic properties. These include the polyphenol brasilin and the tanshinones—TS1, TS2, and TS0 (Figure 5).

### 6.1. Brazilin

Brazilin (7,11b-dihydrobenz[b]indeno[1,2-d]pyran-3,6a,9,10(6H)-tetrol) (Figure 5) is a natural polyphenol and homoisoflavonoid found in *Caesalpinia echinata* (Brazilwood), *Biancaea sappan* (sappanwood), *Paubrasilia echinata*, and *Haematoxylum brasiletto* [391]. It is a precursor of brazilein, an active red dye that represents the oxidized form of brazilin. Brazilin exhibits a variety of biological activities, including anti-inflammatory, hypoglycemic, antihepatotoxic, antibacterial, antioxidant, and antiplatelet aggregation effects [392,393,394,395,396,397,398]. Brazilin was identified through virtual screening as a promising inhibitor of Aβ fibrillogenesis by Du et al., and this potential was validated by multiple experimental analyses [31]. These studies demonstrated that brazilin functions as a dual-action agent, both inhibiting fibril formation with IC_50_ of 1.5 µM and remodeling mature Aβ fibrils. Brazilin was found to redirect Aβ_1-42_ monomers and mature fibrils into unstructured aggregates containing some β-sheet elements, thereby preventing both primary nucleation and fibril-catalyzed secondary nucleation.

In vivo pharmacokinetic studies of brazilin indicate its stability and its capacity to cross the BBB [399,400,401].

To gain atomistic insight into these mechanisms, the authors conducted cMD simulations using 10 brazilin molecules in the presence of a fibrillar Aβ_17-42_ pentamer (Table 4). The simulations revealed that brazilin binds to multiple sites on the Aβ_17-42_ pentamer, preferentially interacting with residues L17, F19, F20, and K28. Strong hydrophobic interactions were observed between the phenyl rings of brazilin and F20, while its hydroxyl groups formed over ten hydrogen bonds with the fibril. Importantly, brazilin disrupted the critical intermolecular salt bridge between D23 and K28—which is essential for fibril stability—by forming hydrogen bonds with K28. In the presence of brazilin, the number of interchain backbone hydrogen bonds was reduced by nearly half, leading to significant fibril destabilization and remodeling, thereby preventing further fibril formation [31].

### 6.2. Tanshinones TS1 and TS2

Tanshinones are lipophilic phenanthrene-based compounds, also classified as diterpenoids, naturally found in the roots of the *Salvia miltiorrhiza* (Danshen or red sage), a traditional Chinese medicinal herb from the Lamiaceae family [402,403]. They possess a broad spectrum of pharmacological activities, including antioxidant, anti-inflammatory, antibacterial, and antitumor effects, and have been widely used in the clinical treatment of cardiovascular diseases such as ischemic and hemorrhagic conditions, atherosclerosis, and hypertension. Owing to their lipophilicity and low molecular weight, tanshinones can cross the BBB. Tanshinones are characterized by poor water solubility and low oral bioavailability [404]. Notably, they exhibit anti-acetylcholinesterase activity and demonstrate significant neuroprotective properties, particularly tanshinone I (TS1) and tanshinone IIA (TS2) (Figure 5). TS2 has been shown to protect against Aβ-induced neurotoxicity, reduce phosphorylated tau levels, and enhance neuronal viability [402]. It mitigates AD risk and suppresses neuroinflammation by downregulating the transcription and expression of genes encoding inducible nitric oxide synthase (iNOS), matrix metalloproteinase-2 (MMP-2), and the transcription factor NF-κBp65 [405]. Experimental studies demonstrated that both TS1 and TS2 inhibit Aβ aggregation, disassemble preformed fibrils, and protect SH-SY5Y cells from Aβ-induced toxicity, with TS1 exhibiting greater inhibitory potency than TS2 [406]. To investigate their mechanism of Aβ inhibition, the authors further performed MD simulations (Table 4). MD simulations showed that TS1 preferentially binds to two main sites: BS A1, comprising I31–M35 on the external surface of the hydrophobic C-terminal β-sheet; and BS A2, consisting of aromatic residues F4–H6 near the N-terminus, more energetically favorable than secondary binding sites BS A3 and A4. Key contact residues for TS1 included F4, H6, I31, G33, L34, and M35. TS2 was found to interact with seven distinct sites (B1–B7) on the Aβ pentamer. Approximately 30% of the binding poses were associated with B1 and B6, both of which involve residue Y10 and overlap with TS1’s BS A1 and A2. The binding affinities at these sites were similar, with key contact residues including F4, H6, Y10, V39, F40, and I41. Based on these findings, the authors proposed that tanshinones inhibit Aβ aggregation through multiple mechanisms: blocking lateral association between aggregates when bound to β-strand groove regions (e.g., I31–M35 and M35–V39), disrupting local secondary structures when interacting with turn or terminal regions, and introducing steric hindrance by stacking TS1 or TS2 dimers/trimers atop the pentameric fibril, thereby creating an energy barrier to further aggregation [406].

**Table 4:** Summarized technical data and key findings from the reviewed MD simulations of natural compounds with three-condensed-ring scaffold. Years reflect advances in technology, computational power, and research outcomes over the last twelve years.

FF/Water Model	Duration per System, ns	Aβ Length/PDB ID/Type (Monomer/Dimer/ (Proto-) Fibril)	Inhibitor *	Main Findings	Year, Ref.
GROMOS96/SPC	3 × 100	Aβ_17-42_/2BEG/pentamer, protofibril	10 brazilin	preferably interacts with L17, F19, F20 and K28; reduces interchain H-bonds; inhibits fibrillogenesis, forming unstructured aggregates; remodels Aβ fibrils via disrupting the salt bridge D23–K28	2015 [31]
CHARMM27/TIP3P	2 × 40 per system	Aβ_1-42_ built from experimental U-shaped Aβ_9-40_ data [85,89]/modelled pentamer	5 and 10 TS1; 5 and 10 TS2	TS1: two primary BS were identified, which are the most energetically favourable; BS A1—at the β-sheet groove at C-terminal (I31–M35); BS A2—near the N-terminus (F4–H6); forms contacts with: F4, H6, I31, G33, L34, M35; TS2: seven BSs (B1–B7), BS1 and BS6, near at Y10, cover ~ 30% of pose population, as BS B1 = BS A1 of TS1; BS6 = BS A2; forms contacts with F4, H6, Y10, V39, F40, I41	2013 [406]
GROMOS 53a6/TIP3P	3 × 100—300 per system	(1) Aβ_1-40_/2LMN/monomer; mAβ_40_ (2) Aβ_1-40_/2LMN/pentamer, protofibril; pAβ_40_ (3) Aβ_1-42_/2BEG/monomer; mAβ_42_ (4) Aβ_1-42_/2BEG/pentamer, protofibril; pAβ_42_	(1) & (3) 2 TS1; (2) & (4) 10 TS1; molar ratio 2:1	(1) binds to four regions—DR, β1, turn, and β2; increases β-sheet content; (3) binds also to DR region; leading to decrease in β-sheet content; (1) & (3) preferred binding residues F4, D7, Y10, K16, F20, K28, and V40; (2) & (4) mainly located in DR; (2); β-sheet content remains unchanged; (4) decreases β-sheet content, accompanied by an increased twist angle and disappearance of the bend region (^37^ GGV ^39^), resulting in reduced structural stability;	2017 [407]

As most acronyms are identical to those in the previous table, only the new ones are listed below: TS1, TS2—Tanshinone 1 and 2; DR—disordered region. * number of molecules is specified when different from 1.

A more detailed MD study of TS1 and its demethylated derivative TS0 (Figure 5) was conducted by Dong and colleagues [407]. The study involved simulations of four systems: monomeric Aβ_1-40_ (mAβ_1-40_) and Aβ_1-42_ (mAβ_1-42_), as well as pentameric Aβ_1-40_ (pAβ_1-40_) and Aβ_1-42_ (pAβ_1-42_), using crystallographic structures that differed conformationally from those employed by Wang et al. [406], resulting in distinct observations (Table 4). In the mAβ_1-40_ system, TS1 interacted with residues in the disordered region (DR), β1 strand, turn, and β2 strand, leading to a 10% increase in β-sheet content and overall structural stabilization. In contrast, in the mAβ_1-42_, TS1 primarily bound within the DR and caused a reduction in β-sheet content. Across both monomer systems, the most frequent contact residues were F4, Y10, K16, F20, K28, and V40, with D7 uniquely present in mAβ_1-42_. In the pentameric systems (pAβ_1-40_ and pAβ_1-42_), TS1 predominantly localized to the DR and partially to salt-bridge regions. In pAβ_1-40_, TS1 preserved β-sheet content compared to the control, indicating that fibril stability remained largely unaffected. However, in the pAβ_1-42_, TS1 reduced β-sheet content, increased the fibril twist angle, and led to the disappearance of the bend region (residues ^37^ GGV ^39^), resulting in decreased stability and a conformation more closely resembling Aβ_1-40_. Binding within the DR was found to shift the β1 region, contributing to fibril twisting around its axis. This, in turn, disrupted β-sheet integrity, reduced fibril stability, and hindered lateral association of Aβ aggregates, thereby inhibiting fibril growth.

TS0, a demethylated analog of TS1 synthesized by Liu et al. [408], exhibited distinct behavior in MD simulations [407]. It consistently reduced β-sheet content in both mAβ_1-40_ and mAβ_1-42_, primarily interacting with the peptides’ disordered regions. Key contact residues included D1–D7, Y10, E11, K16, E22, D23, K28, and V40, with D7 and E11 being uniquely conserved in mAβ_1-42_. This behavior contrasts with that of TS1, suggesting that demethylation significantly alters the compound’s interaction profile and structural effects on Aβ peptides. In pentameric systems, TS0 is also localized mainly to the DR (albeit to a lesser extent than TS1) and more prominently to salt-bridge regions. In pAβ_1-40_, TS0 slightly increased β-sheet content, whereas in pAβ_1-42_ systems, it reduced β-sheet content, increased twist angle, and caused disappearance of the bend region (^37^GGV^39^), mirroring and enhancing the effects seen with TS1. Notably, TS0 induced a more pronounced reduction in β-sheet content and more effectively promoted fibril twisting in pAβ_1-42_, indicating superior disaggregation potential compared to its methylated counterpart.

## 7. Other Natural Compounds Inhibiting Aβ

**Figure 6:**
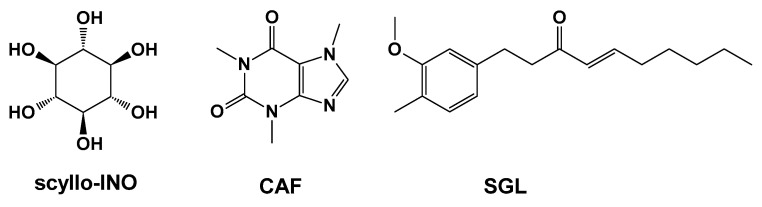
Natural compounds investigated via MD simulations for their inhibitory effects on Aβ aggregation.

In this section, we discuss additional natural compounds that have been investigated for their anti-amyloid mechanisms using MD simulations. These include *scyllo*-inositol (*s*INO), caffeine (CAF), and 6-shogaol (SGL), as illustrated in Figure 6.

### 7.1. Scyllo-Inositol (sINO)

*Scyllo*-inositol, also known as scyllitol, cocositol, or quercinitol, is a natural sugar alcohol and one of nine stereoisomers of inositol (cyclohexanehexol). It occurs naturally in plants, certain bacteria, mammalian tissues, and is particularly abundant in coconut milk [409,410,411]. Inositol stereoisomers are involved in a range of physiological processes, including gene expression regulation, fat metabolism, membrane potential maintenance, intracellular calcium signaling, cytoskeletal organization, telomere length regulation, and insulin secretion [412]. *s*INO is one of four physiologically active inositol stereoisomers, along with *myo-*, *epi-*, and *chiro*-inositol. In the human brain, *s*INO concentrations—measured by NMR—are approximately 0.35 mM in white matter, 0.4 mM in gray matter, and 0.5 mM in the cerebellum. In contrast, *myo-*inositol, the most abundant stereoisomer, is present at around 5 mM across various cortical regions [413,414]. McLaurin et al. demonstrated that in vitro, *s*INO, along with *epi-* and *myo-*inositol, induced a conformational shift from random coil to β-sheet structures in Aβ_1-42_ monomers; however, these β-sheet-rich complexes did not proceed to fibrillization [415]. Additionally, *s*INO significantly reduced oligomer-induced cytotoxicity, with both aggregation inhibition and cytoprotective effects showing stereoisomer-specific activity (*scyllo*- > *epi*- > *myo*-). The authors further investigated the in vivo efficacy of *epi-* and *scyllo*-inositol in a transgenic mouse model of AD [416]. *s*INO treatment significantly improved cognitive and behavioral outcomes, while reducing brain Aβ levels, amyloid plaque burden, synaptic loss, glial activation, and mortality—effects observed regardless of whether treatment began in presymptomatic or symptomatic stages. In another study, *s*INO dose-dependently neutralized the inhibitory effects of soluble Aβ oligomers on long-term potentiation in mouse hippocampal slices. Oral administration prevented Aβ-induced cognitive deficits and was shown to have good bioavailability, cross the BBB, and cause no adverse effects on hippocampal synaptic function [417]. Notably, *s*INO displayed a favorable safety profile, showing no toxicity even at concentrations tenfold higher than endogenous levels, supporting its potential for therapeutic use at elevated doses [417,418]. However, despite these promising effects, a phase 2A randomized, double-blind, placebo-controlled clinical trial in patients with mild to moderate AD reported adverse effects—including falls, depression, confusion, and some severe outcomes such as death—at higher doses of *s*INO [419]. As a result, the trial continued at lower doses, but no significant improvements in cognitive or functional outcomes were observed compared to placebo.

A comprehensive study investigating the binding mechanism of *s*INO to monomers and aggregates of Aβ_16-22_ using MD simulations was conducted by Li and Pomès (Table 5) [420]. The authors examined three distinct Aβ morphologies: monomers, disordered oligomers, and β-oligomers (protofibrillar-like aggregates).

MD simulations of monomeric Aβ_16-22_ revealed that *s*INO binds weakly and reversibly, with minimal dependence on molar ratio. Binding was dominated by nonpolar interactions, primarily involving phenylalanine (F) residues, while hydrogen bonds were mainly formed with glutamate (E) and, to a lesser extent, lysine (K). Simulations of four dispersed monomers resulted in the spontaneous formation of disordered oligomers with predominantly random coil conformations, irrespective of *s*INO presence, concentration, or ratio. *s*INO did not significantly influence aggregation kinetics, hydrophobic packing, or oligomer morphology in these systems. In the case of the pre-formed β-oligomers, *s*INO primarily bound to the surface of the aggregates. It interacted with the nonpolar regions of F and K residues, as well as the charged groups of K and E, but did not penetrate the β-sheet core or induce disaggregation. At higher concentrations, *s*INO molecules formed clusters that adsorbed onto the surface of the β-oligomers. Based on these findings, the authors proposed that *s*INO may inhibit fibril formation by coating β-sheet surfaces, thereby disrupting lateral stacking and blocking further fibrillization.

### 7.2. Caffeine (CAF)

Caffeine is the most widely consumed psychoactive substance globally. As a central nervous system stimulant and a natural purine alkaloid (methylxanthine), it is found in coffee, tea, cacao, and guarana beans. Caffeine is well known for its diverse pharmacological and physiological effects, including cardiovascular, respiratory, and renal actions, as well as its antioxidant and anti-inflammatory properties [422,423,424,425]. It also modulates mood, memory, alertness, and enhances both physical and cognitive performance. CAF has demonstrated neuroprotective effects against Aβ-induced neurotoxicity in SH-SY5Y cells, primarily through inhibition of adenosine and NMDA (N-methyl-D-aspartate) receptors and activation of ryanodine receptors [426]. In vivo studies suggest that its protective actions are mediated by enhanced Aβ clearance via induction of P-glycoprotein in the brain [427]. In D-galactose-treated rats, caffeine improves memory and reduces oxidative stress, inflammation, apoptosis, and neurodegeneration [428]. Moreover, in Alzheimer’s disease mouse models, caffeine has been shown to lower Aβ levels in plasma and brain tissue—particularly in the hippocampus and entorhinal cortex—reduce Aβ production, and improve cognitive function [429,430,431].

While direct experimental evidence for caffeine’s inhibition of Aβ aggregation remains limited, Gupta and Dasmahapatra explored its effects on an Aβ_17-42_ protofibril using cMD simulations [421]. Their findings revealed that caffeine destabilizes the protofibril by reducing β-sheet content, particularly in the β2 region, converting it into helices and disordered structures such as random coils, bends, and turns. This structural disruption is accompanied by a loss of hydrogen bonds and hydrophobic interactions between monomers, as well as the breakdown of the critical D23–K28 salt bridge.

A concurrent in vivo study investigated coffee consumption in relation to amyloid pathology and other Alzheimer’s disease (AD) markers in 411 older adults (282 cognitively normal, 129 with mild cognitive impairment) [432]. Lifetime intake of ≥2 cups/day was significantly associated with lower cerebral Aβ positivity, even after adjusting for confounders, whereas neither lifetime nor current coffee intake was linked to hypometabolism, atrophy in AD-signature regions, or WMH volume. These findings suggest that higher lifetime coffee intake may help reduce AD risk by limiting cerebral amyloid deposition.

A recent study of 4394 cognitively normal older adults examined the relationship between caffeine consumption and brain amyloid positivity [433]. Overall, no significant association was found. Subgroup analysis, however, revealed that caffeine intake was inversely associated with amyloid positivity in males but not in females, and this association was unaffected by age or APOE genotype. Additionally, varying levels of caffeine intake showed no link to amyloid positivity. These findings suggest a potential sex-specific effect, warranting further studies to clarify the mechanisms underlying caffeine consumption and brain amyloid deposition.

### 7.3. 6-Shogaol (SGL)

*6*-shogaol is the major bioactive compound derived from dried ginger rhizomes (*Zingiber officinale* Roscoe), formed through the dehydration of *6*-gingnerol. It is well known for its anti-inflammatory, antioxidant, antiemetic, anticancer, and analgesic activities [434,435,436,437]. In addition to these effects, SGL exhibits both neuroprotective and neurotrophic properties, including the reduction in reactive oxygen species (ROS) and the enhancement of brain-derived neurotrophic factor (BDNF) levels [438]. Its neuroprotective activity has also been linked to increased expression of choline acetyltransferase and choline transporter, mediated via BDNF upregulation in H_2_O_2_-treated HT22 hippocampal neuronal cells [439]. SGL has demonstrated improvements in cognitive impairment by reducing microgliosis and astrogliosis, and by increasing nerve growth factor (NGF) levels in Aβ- and scopolamine-induced animal models of dementia [440]. In vitro and in vivo studies showed that 6-shogaol inhibits the CysLT1R/cathepsin B pathway, reducing Aβ deposition and improving behavioral deficits in APPSw/PS1-dE9 mice [441]. Pretreatment with 6-gingerol significantly improved cell viability and reduced apoptosis in Aβ_1-42_-treated PC12 cells [442]. It also lowered intracellular ROS, MDA, NO production, and LDH leakage, while enhancing SOD activity. Additionally, 6-gingerol upregulated p-Akt and p-GSK-3β protein levels [442]. These findings suggest that 6-gingerol protects against Aβ_1-42_–induced apoptosis by mitigating oxidative stress and inflammation, inhibiting GSK-3β activation, and promoting Akt signaling, thereby exerting neuroprotective effects.

In a previously discussed in silico study (Section 4.1, Section 4.5, Section 4.6, and Section 5.6), ensemble docking screening followed by MD simulations targeting S-shaped Aβ_11-42_ fibrils identified SGL as a potential Aβ aggregation inhibitor [206]. Similarly to OLUE, SGL was found to intercalate between adjacent peptide chains, leading to destabilization of the fibril structure (Table 5). It reduced β-sheet content, fibril order, and inter-chain interaction surfaces. SGL primarily interacted with the H14–G25 region. Notably, during the MD simulation, SGL detached from its binding site, suggesting a transient or unstable interaction.

## 8. Discussion

Given that the natural compounds were grouped according to structural similarities, this section provides an overview of their anti-amyloid behavior, emphasizing shared mechanisms of inhibition, key contact residues, and intermolecular interactions. However, consistency across MD simulations is limited, as different studies have investigated diverse Aβ_1-42_ forms (monomeric, dimeric, U-, S-, and LS-shaped fibrils) along with variation in peptide/inhibitor molar ratios. Consequently, the following analysis is selective rather than comprehensive. The summarized data are presented in Table 6 to enhance clarity and facilitate cross-study comparison.

The final uncategorized group of compounds was excluded from this comparison, as *s*INO and CAF showed no detectable anti-amyloidogenic effects at safe doses [419,432], and the SGL–S-shaped fibril complex proved unstable in MD simulations. Their relatively low molecular weights and limited conjugated ring systems—unlike the more complex scaffold 1, flavonoid, or condensed-ring scaffolds—likely reduce their capacity for stable binding to Aβ structures or might require higher concentrations to exert activity.

Only CCN and RESV from the first compound group (scaffold 1) have been simulated on monomeric Aβ. As shown in Table 6, CCN moves around the peptide and interacts through multiple nonspecific contacts [199,200,201,202,210], whereas RESV significantly reduces β-sheet content and destabilizes the α-helix by forming H-bonds with Q15 and D23 and altering the M35–I31 distance [213]. These differences likely arise from RESV’s shorter linker, which confers a more compact structure, and from variations in the number and position of phenolic OH groups.

Compounds from the flavonoid scaffold group studied on monomeric Aβ include EGCG, MYR, and MOR. EGCG inhibits β-sheet formation in a dose-dependent manner, interacting with key residues such as F4 (hydrophobic contacts), R5, F19, F20 (H-bonding and hydrophobic contacts), K28, G29, L34, M35, V36, G37, and I41 [302]. In contrast, MOR shows minimal effect at low molar ratios; however, at higher ratios it covers Aβ_1_**_-_**_42_, restricting its collapse and α-helix/β-sheet interconversion, thereby significantly altering both secondary and tertiary structures—possibly reflecting the early formation of “off-pathway” aggregates [297]. The stronger inhibitory effect of EGCG may be attributed to its additional D ring, three extra hydroxyl groups, and unsaturated C ring compared to MOR (Figure 4).

When MOR and MYR were studied on U-shaped fibril-extracted monomer, similar effects were observed: both penetrated the Aβ core, reduced β-sheet content, induced bends or coils, and destabilized the monomer. Their dominant interactions involved H-bonds with CO and NH groups of Aβ. At higher molar ratios, MOR formed self-clustered Aβ–MOR complexes [30,311].

From the condensed-ring scaffold group, only TS1 was studied on monomeric Aβ. TS1 binds to the DR region, decreases β-sheet content, and interacts with residues F4, D7, Y10, K16, F20, K28, and V40 [407].

The U-shaped fibril is the most extensively studied structure, as shown in Table 6. A shared inhibitory mechanism among scaffold 1 compounds (CCN, gx-50, RESV, and OleuA) involves binding within the concave region of the β-strand–loop–β-strand motif, leading to fibril destabilization through β-sheet reduction and disruption of the critical D23–K28 salt bridge [205,210,212,213,214]. While CCN and gx-50 primarily distort peripheral chains, RESV promotes the formation of unordered, non-toxic aggregates and reduces both α-helical and β-sheet content, thereby disrupting fibrillar organization. In contrast, OleuA induces complete fibril disassembly. Additionally, CCN has been shown to bind directly to the fibril surface, further disrupting the D23–K28 salt bridge [204].

MYR and MOR, from the flavonoid scaffold group, remodel rigid, toxic U-shaped protofibrils into expanded, fragile, and nontoxic amorphous aggregates. They achieve this by reducing the number of interchain hydrogen bonds, while maintaining β-sheet structures but significantly disrupting the rigid “steric-zipper” motif [30].

The distinct position of ring B in GEN may explain its different mode of disrupting U-shaped fibrils—it stabilizes the protofibril while reducing β-sheet content. GEN preferentially localizes at the C-terminal β-sheet groove near G33 and M35, forming contacts with L17, F20, E22, I31, G33, M35, and V39 [314].

Compounds from the condensed-ring scaffold (TS1, TS2, and brazilin) exhibit multiple mechanisms of Aβ aggregation inhibition, characterized by several binding sites. TS1 and TS2 interact at two main sites: A1, located in the β-sheet groove on the external surface of the hydrophobic C-terminal β-sheet (I31–M35), and A2, near the N-terminus (F4–H6) [406]. Brazilin preferentially interacts with residues L17, F19, F20, and K28, forming strong hydrophobic contacts between its phenyl rings and F20, while its hydroxyl groups establish multiple H-bonds [31]. This results in reduced interchain backbone H-bonds and disruption of the D23–K28 salt bridge. In another simulation, TS1 was found to primarily localize in the disordered region (DR), inhibiting fibril growth by disrupting β-sheet integrity, reducing fibril stability, hindering lateral association of Aβ aggregates, increasing the twist angle, and eliminating the bend region (^37^ GGV ^39^) [407].

The effects of natural compounds on S-shaped fibrils have been less extensively studied. Only CCN, PCT, and OLEU from scaffold 1, as well as GOS and amentoflavone-type biflavonoids from the flavonoid scaffold, have been simulated. Interestingly, a study by Muscat et al. found that CCN, PCT, and GOS insert into a pocket within the fibril, disrupting the ordered structure, decreasing β-sheet content, reducing fibril order and inter-chain interaction surfaces, and binding to the E11–F19 and I32–L24 regions [206]. OLEU binds between adjacent peptide chains, leading to significant fibril destabilization, preferentially interacting with residues V18–V24 and N27–I31, resulting in interchain disruption and a marked reduction in both β-sheet content and overall fibril order In another study, CCN was shown to break down all β-sheets within the ^12^VHHQKLVFF^20^ domain of the outermost peptide, causing partial fibril dissociation [207].

Amentoflavone-type biflavonoids induce conformational changes in the fibril, promoting disaggregation [315]. They form π–π interactions with F4, H6, Y10, H13, and H14, and significantly reduce β-sheet content, with key hydroxyl groups at the R2 and R3 positions forming H-bonds with the peptide backbone.

The LS-shaped fibril structure has been investigated only for EGCG in two independent studies, whose results partially overlap in the identified binding sites (BSs) (Table 6) [308,310]. Both studies independently identified two similar BSs: one located on the surface near the N-terminus (D1–G9) and the other near the C-terminus (K28–A42). Additional BSs were also reported, including one within the fibril core at F19 and another involving residues E11 and H13 [310]. Mechanistically, EGCG disrupts key stabilizing interactions, including the K28–A42 salt bridge, increases the kink angle around Y10, reduces hydrogen bonding in the H6–E11 segment while enhancing it in the E11–H13 region, and engages in multiple interactions such as hydrogen bonds with E11, π–π stacking with H14 and Y10, and a cation–π contact with K28 [308].

As different authors have emphasized distinct aspects of MD trajectories, a rigorous structural comparison of compounds within and across scaffolds can only be achieved through future exhaustive simulations conducted under standardized conditions. Such studies should encompass all natural compounds (or groups, as classified in this review) and employ consistent parameters with respect to Aβ form (monomeric, dimeric, and U-, S-, and LS-shaped fibrils), inhibitor-to-peptide molar ratio, and simulation duration. A systematic approach of this kind would enable a clearer understanding of how structural differences modulate inhibitory mechanisms, facilitate the identification of key molecular features underlying anti-amyloid activity, and inform the rational optimization of compound structures to enhance efficacy while addressing inherent limitations in stability, solubility, and bioavailability.

## 9. Key Challenges and Feature Remarks

Identifying Aβ inhibitors through structure-based drug design (SBDD) using molecular docking and MD simulations remains a significant challenge due to complexity of fibril formation and the existence of multiple binding sites. Virtual screening can aid in identifying potential inhibitors, which can then be refined through MD simulations and validated experimentally. Effective inhibitors often act by disrupting the β-sheet structures that stabilize amyloid fibrils. Both structure-based and ligand-based drug design strategies play important roles in the hit-to-lead optimization process for Aβ inhibitors.

Mechanistically, Aβ inhibition generally falls into three main categories: (1) blocking early-stage monomer interactions to prevent dimer formation, (2) interfering with the growth of small oligomers, and (3) destabilizing existing oligomers to redirect aggregation toward non-toxic pathways. An additional, though less commonly studied mechanism, involves blocking the lateral association of fibrils, thereby inhibiting fibril growth.

This review highlights the powerful applications of MD simulations in the identification and mechanistic characterization of natural compounds as potential anti-amyloid agents. For example, MD facilitated the identification of PCT, whose anti-aggregation activity was later confirmed with an IC_50_ of 0.48 µM. Beyond this, PCT also demonstrates antioxidant and anti-acetylcholinesterase activity, protects against Aβ-induced neurite fragmentation, and suppresses Aβ-induced neuronal cell death, making it a promising multitarget agent with pleiotropic effects against AD.

MD simulations also strongly complement in vitro and in vivo approaches in the development of natural Aβ inhibitors. While MD provides atomistic-level insights into binding interactions, conformational changes, and aggregation pathways, in vitro methods such as atomic force microscopy (AFM), NMR, Thioflavin T fluorescence assays, and cell viability assays validate these mechanisms by directly visualizing fibril morphology, tracking aggregation kinetics, and assessing cytotoxicity. Importantly, MD findings often align with experimental results, helping to explain fibril disaggregation mechanisms for compounds such as CCN, RA, lemairamin (wgx-50), RESV, OLEU, EGCG, MYR, QUER, MOR, GEN, amentoflavones, brazilin, TS1, and TS2. Conversely, MD can also confirm the absence of anti-amyloid effects, as observed with *s*INO. Simulations further clarify distinct inhibition mechanisms, such as those predicted for TS1 and its demethylated derivative, TS0, the latter expected to display even stronger inhibitory activity—though this requires experimental validation.

At the same time, experimental findings refine and calibrate MD simulations by providing structural and functional benchmarks, thereby improving predictive accuracy.

This review also discusses the limitations and occasional discrepancies of MD predictions, such as those observed with CAF and OleuA, underscoring the need for further refinement. Additionally, compounds including MOR, GOS, and TS0 still await experimental confirmation of their MD-predicted anti-amyloid activities.

Carefully designed MD simulations, followed by in vitro and in vivo validation, are essential for elucidating these mechanisms and advancing drug development. As previously discussed [23], the structural complexity and morphological diversity of Aβ fibrils present further challenges in understanding how small molecules inhibit amyloid aggregation. Notable hurdles include selecting appropriate force fields and water models that faithfully represent fibrillization while balancing computational costs. For MD studies of anti-amyloid inhibitors to be meaningful, the following considerations are critical: (1) use full-length Aβ peptides (monomers or small oligomers); (2) simulate diverse fibril morphologies (U-, S-, SL-shaped) to capture structural variability; (3) carefully select and, if possible, compare multiple force fields; (4) match water models with the chosen force field to ensure consistency; (5) apply inhibitor-to-peptide ratios that reflect experimental and physiological conditions, testing multiple ratios as needed; (6) include appropriate controls (positive, negative, and inhibitor-free) under identical simulation conditions, including physiological pH; (7) ensure simulation times are sufficient to observe relevant structural changes; (8) analyze stabilizing interactions and changes in β-sheet content to assess the inhibition mechanism; (9) perform multiple replicates with varied initial velocities to produce robust, statistically reliable results.

While MD and experimental studies underscore the anti-amyloidogenic potential of natural compounds, their progression toward drug approval depends critically on pharmacokinetic properties, including solubility, stability, BBB permeability and bioavailability. Most of the reviewed natural compounds—CCN, RA, RESV, PCT, OLEU, EGCG, MYR, QUER, MOR, GEN, AMF, brazilin, and tanshinones—generally exhibit poor oral bioavailability due to low solubility, rapid metabolism, limited absorption, and/or physiological instability. To address these challenges, various strategies have been explored, including chemical modifications, nanoparticle-based delivery systems, inclusion complexes, and other formulation approaches to enhance stability, absorption, BBB penetration, and overall therapeutic potential. Despite these limitations, some compounds, such as lemairamin and brazilin, demonstrate promising pharmacokinetic profiles, including the ability to cross the BBB, supporting their potential for central nervous system-targeted therapies.

In our previous publication, we reviewed endogenous compounds and repurposed drugs studied as Aβ inhibitors using MD simulations [23]. In this review, we expand upon that work by focusing on a variety of natural compounds that exhibit anti-amyloid activity, also explored through MD approaches. Together, these insights can support the rational design of novel Aβ inhibitors and inform best practices for MD simulations aimed at uncovering their mechanisms of action.

By offering atomistic insights into binding modes, conformational changes, and aggregation pathways, MD simulations bridge critical gaps that cannot be fully captured by in vitro or in vivo experiments alone. These mechanistic details not only clarify experimental ambiguities but also enable prioritization of candidate molecules before entering costly preclinical pipelines.

Looking ahead, integrating MD with structural biology, pharmacokinetics, and systems-level modeling, alongside advanced experimental tools, can establish a robust discovery pipeline for anti-amyloid therapeutics. The synergy between computational and experimental strategies creates a powerful framework: MD generates predictive hypotheses, in vitro studies validate molecular interactions, and in vivo models confirm therapeutic relevance.

This iterative approach can accelerate translation, reduce development costs, and guide the rational design of natural-product-derived therapeutics. Ultimately, the combined use of MD and experimental validation holds strong promise for advancing biologically relevant natural compounds toward clinical development, offering new opportunities for Alzheimer’s disease therapy.

## 10. Conclusions

This review demonstrates that molecular dynamics (MD) simulations are a powerful tool for identifying and mechanistically characterizing natural compounds as potential anti-amyloid agents. MD provides atomistic insights into binding interactions, conformational changes, and aggregation pathways, which, when combined with in vitro and in vivo validation, clarify fibril inhibition and disaggregation mechanisms. Compounds such as PCT, EGCG, RA, OLEU, CCN, and others show promising multitarget anti-amyloid activities, while MD also identifies compounds with limited efficacy, such as sINO. Experimental findings refine MD predictions, improving their accuracy, though discrepancies remain for compounds like CAF and OleuA. Some molecules, including MOR, GOS, and TS0, still require experimental confirmation. Together, MD and experimental integration accelerate the rational design of natural-product-derived Aβ inhibitors and support future preclinical development.

## Figures and Tables

**Table 1:** Enhanced sampling methods in molecular dynamics (MD), summarizing their principles, advantages, and limitations in overcoming the timescale and sampling constraints of conventional MD.

Category	Method	Principle	Advantages	Limitations/Challenges	Refs.
Generalized-ensemble methods	Simulated Tempering (ST)	Involves the initial calculation of probability weight factors	Improves sampling efficiency	Computationally expensive weight calculations	[127,128]
	Replica Exchange Method (REM) (Parallel Tempering)/Replica Monte Carlo/Multiple Markov Chain	Uses predefined probability weight factors; runs multiple non-interacting replicas at different temperatures with periodic exchanges via Metropolis criterion	Avoids weight calculation; effective barrier crossing	Very computationally demanding; needs parallel computing	[84,85,124,129,130,131,132,133,134]
	Replica Exchange MD (REMD)	REM adapted to MD (accounts for momenta/velocities)	Powerful for biomolecular sampling	Extremely costly for large/complex systems; needs parallel computing	[135]
	Simulated Tempering Distributed Replica (STDR)	Optimized for distributed/heterogeneous computing	Minimal initial simulation; unaffected by replica synchronization or a fixed number of temperatures	Same risk of non-physical transitions causing a loss of dynamic information as all generalized-ensemble methods	[121,136]
Coarse-graining/reduced resolution	Coarse-Grained (CG) Modeling (e.g., MARTINI, implicit solvent)	Groups atoms into single particles	Longer simulations possible; reduced degrees of freedom	Reduced accuracy vs. atomistic MD	[137,138]
Biasing potential methods	Conformational flooding	disrupt local energy minima, enabling the system to escape energy wells more efficiently	Accelerated transitions; lowered free energy barriers	Requires fine-tuning	[139,140]
	Metadynamics	Adds Gaussian bias to Hamiltonian; pushes system away from visited states	Efficient exploration of new conformations	Choice of parameters critical	[141,142,143,144,145]
	Umbrella Sampling	Applies bias to specific regions	Focused sampling of rare events	Needs prior knowledge of reaction coordinate	[146,147]
	Accelerated MD (aMD)	Modifies the system’s potential energy landscape; raises energy minimum of the wells, lowering barriers between states	No predefined reaction coordinate; long-timescale accessible	Accuracy decreases in large systems; bias artifacts	[148,149]
	aMD Variants (replica exchange aMD (REXAMD) and selective aMD	Refinements of aMD (replica-based, region-specific)	Balances accuracy and efficiency	Still under refinement for large/complex systems	[150,151]

**Table 2:** Summarized technical data and key findings from the reviewed MD simulations. While most studies employed cMD, some utilized alternative approaches, which are indicated accordingly. The years are provided to help readers follow the progression of technical advances, computational power, and the corresponding research outcomes reported over the past twelve years.

FF/Water Model	Duration per System, ns	Aβ Length/PDB ID/Type (Monomer/Dimer/ (Proto-) Fibril)	Inhibitor *	Main Findings	Year Ref.
GROMOS96 53a6/SPC	500	Aβ_1-42_/1IYT/generated dimer	CCN	reduces β-sheet structure; forms π–π stacking with F19; travels around Aβ, frequently close to L34	2012 [199]
Amber99SB/TIP3P	200	Aβ_1-42_/1Z0Q/monomer	(1) CCN (2) CCN + Cu^2+^ located at (i) H13 and (ii) H13δ	(1) forms H-bonds with K16, F19, and F20; (2) (i) the secondary structure is altered, as CCN interacts with the F4–D7 α-helix and turn region Y10–V12 initially, followed by interaction with the C-terminal unfolded region I32–A42; Cu^2+^ interacts with D7 and D23; (ii) CCN stabilizes the Y10–D23 α-helix and prevents formation of β-sheet structures at the C-terminal; interacts with Y10, H13, L17, F20, V24, K28, and I32; forms H-bonds with S8, G9, Y10, and K28; Cu^2+^ initially was chelated by CCN and later interacts with D7	2015 [200]
Amber ff14SB/TIP3P	1000	Aβ_1-42_/1IYT/monomer	CCN	stabilizes the monomer structure; increases the propensity of α -helices and decreases the propensities of β-turns; numerous diverse interactions—including H-bonds (with F4, H13, E11, G33, and L24), hydrophobic contacts (with E3, F4, R5, H6, D7, Y10, and H13), π–π (with F4, Y10, H13, H14, and F20), and cation–π (with R5 and K16)—across all regions of the Aβ peptide	2021 [201]
Amber ff14SB/TIP3P	1000	Aβ_1-42_/1IYT/12 monomers	12 CCN 36 CCN	contacts the ensemble of monomers via numerous interactions—hydrophobic, H-bonds, with F4, Y10, F19, and F20; and cation–π with R5, K16, and K28; decreases inter-peptide contact number; increases peptide disorder; decreases peptide flexibility	2020 [202]
REST/GROMOS 54a7/SPC	100–800	Aβ_1-40_/2M9R/1, 2, and 3 monomers	CCN in ratio 1:1	decreases β-sheet, increases turn and helical structures; hydrophobic contacts with the CHC of Aβ	2020 [203]
OPLS_2005 (Desmond)/TIP3P	150	Aβ_17-42_/2BEG/pentamer, protofibril	CCN	binds to the C-terminal residues; contacts with F19 (π–π stacking), and with E22, M35, and G37 (H-bonds); destabilizes the peripheral chains; results in highly distorted distances between D23 and K28 in all chains; disturbs the protofibril structure	2015 [204]
CHARMM27 * (NAMD2.9)/TIP3P	100	Aβ_17-42_/2BEG/pentamer, protofibril	CCN	reduces β-sheet structure; increases unorganized regions of the outermost chains; decreases number of intermolecular backbone H-bonds; could lead to destruction of oligomers	2017 [205]
Amber99-ILDN/TIP3P	150	Aβ_11-42_/2MXU/pentamer, fibril	CCN	binds to E11–F19 and I32–L24 areas; affects the whole peptide conformation; decreases β-sheet content, fibril order and inter-chain interaction surface	2020 [206]
CHARM36/TIP3P	100	Aβ_11-42_/2MXU/hexamer, fibril	CCN	partial dissociation of the fibril structure; breakdown of all β-sheets within the ^12^ VHHQKLVFF ^20^ residue domain of the outermost peptide	2020 [207]
DMD + CGM/PRIME20/implicit solvent effects	50,000	Aβ_17-36_/NA/ (1) monomer (2) 8 monomers (3) preformed protofilament (octamer) (4) preformed protofilament (octamer) + 8 monomers	(1) 10 CCN (2) 30 CCN (3) 30 CCN (4) 30 CCN	(1) interacts with L17, F19, F20, A21, I31, I32, L34, and M35 (2) prevent fibrilization by forming disordered complexes (3) binds, but cannot disrupt protofilament structure (4) slows the rate of elongation process	2017 [208]
REMD/GROMOS 57a7/SPC	50 per replica	Aβ_1-40_/2LMN/decamer, protofibril	CCN	primary binds to β-2 BS, and moderate to strong binding to other BSs—β-1, Elbow (loop β-1 and β-2 ^22^EDVGSN^27^), Over (top of the protofibril), and C-terminal; disrupts the secondary structure of the double-layer protofilament; perturbs secondary structure	2018 [209]
GROMOS96 53a6/SPCE/360 K	(1) 200 (2) 100 (3) 100	(1) Aβ_1-42_/1IYT/monomer (2) Aβ_1-42_/1IYT/24 monomers (3) Aβ_17-42_/2BEG/25-mer, fibril	(1) 1 and 4 CCN (2) 2, 5, 19, 77, and 308 CCN (3) 4 and 30 CCN	(1) 1:1 ratio—nonspecific interactions, CCN moves around Aβ; multiple contacts with F, L, V, A, and I; 1:4 ratio—disrupts the β-sheet and increases random coil content (2) forms coarse and disordered structures, lacking β-sheets; at high concentrations—initially self-aggregates before incorporating with Aβ to coarse-grained structures (3) highly specific interactions—individual or stacked CCN molecules that deposit parallel- (predominant) or perpendicular to the fibril axis; enter the fibril hydrophobic core by the open ends or bind to the loop region between two β-strands	2022 [210]
REMD/GROMOS 57a7/SPC	50 per replica	Aβ_1-40_/2LMN/decamer, protofibril	RA	preferably binds to N-terminal and β-1 BS; most stable complexes are at β-2 and β-1 BSs; interacts with M35, G33, and I31 at β-2, and with K16, V18, and F20 at β-1; disrupts the secondary structure of the double-layer protofilament	2018 [209]
RPMD/AMBER parm14SB/TIP3P	100 per replica	Aβ_16-22_/NA/monomer	(1) 1 RA (2) 4 RA	(1) and (2) bind to E22 and K16 via H-bonds, disrupting critical K16–E22 contact, and thus inhibiting oligomerization and preventing the formation and stabilization of cross-β-sheet structure	2020 [211]
AMBER ff03/TIP3P	150 several repeats at 300 and 320 K	Aβ_17-42_/2BEG/pentamer, protofibril	wgx-50	the most probable BS is located inside the U-shaped β-strand-loop-β-strand motif; destabilizes the protofibril; increases the distances between peptides; diminishes the number of the interchain backbone H-bonds; extends the hydrophobic contact distances between A21 and V36; partially disrupts the D23–K28 salt bridges	2015 [212]
DMD + CGM/PRIME20/implicit solvent effects	50,000	Aβ_17-36_/NA/ (1) monomer (2) 8 monomers (3) pre—formed protofilament (octamer) (4) pre + formed protofilament (octamer) + 8 monomers	(1) 10 RESV (2) 30 RESV (3) 30 RESV (4) 30 RESV	(1) strongly binds aromatic amino acids; forms contacts with L17, F19, F20, A21, I31, I32, L34, and M35 (2) prevents fibrilization, forming disordered complexes (3) disrupts the protofilament structure by forming a disordered oligomer (4) stops elongation of protofilament	2017 [208]
GROMOS96/SPC	100	(1) Aβ_1-42_/1IYT/monomer (2) Aβ_17-42_/2BEG/pentamer, protofibril	RESV	significantly reduces the extended β-sheet of both systems; (1) forms H-bonds with Q15 and D23; contacts with V12, H13, Q15, K16, F19, F20, D23, V24, N27, A30, I31, and L34; prevents monomeric aggregation; alters the distance between M35 and I31, leading to destabilization of α-helical structure (2) H-bonds with F19, F20, A21, V36, G37, and G38; interacts with F19, F20, A21, L34, M35, V36, G37, G38, and A40; destabilizes fibrils, leading to unordered non-toxic aggregates	2023 [213]
Amber99-ILDN/TIP3P	150	Aβ_11-42_/2MXU/pentamer, fibril	PCT	disrupts the fibril by inserting into a pocket formed by the S-shaped fibril; induces conformational disorder of the entire fibril structure; reduces β-sheet content and inter-chain interaction surfaces	2020 [206]
Amber99-ILDN/TIP3P	150	Aβ_11-42_/2MXU/pentamer, fibril	OLEU	destabilizes the whole fibril as intercalates between adjacent peptide chains; reduces β -sheet content and inter-chain interaction surfaces; contacts V18–V24 and N27–I31	2020 [206]
OPLS/TIP3P	5000 (5 µs)	Aβ_1-42_/2BEG/pentamer, protofibril	OleuA	strongly destabilizes and perturbs preformed fibrils, leading to their complete disassembly; disrupts the critical D23–K28 salt bridge; increases interchain distances while reducing the number of inter-backbone hydrogen bonds; causes a dramatic reduction in β-sheet content accompanied by an increase in unstructured peptide conformations; primarily targets residues L17–D23, K28, and I31–L34	2020 [214]

Acronyms: DMD—discontinuous molecular dynamics; CGM—coarse-grained model; SPC—imple point charge water model, SPCE—extended simple pint charge water model, TIP3P—transferable intermolecular potential 3-point water model; REMD—replica exchange molecular dynamics; REST—replica exchange solute tempering; RPMD—replica permutation molecular dynamics; CCN—curcumin; RA—rosmarinic acid; wgx-50—lemairamin (gx-50); RESV—resveratrol; PCT—piceatannol; OLEU—oleuropein; OleuA—oleuropein aglycone; BS—binding site; NA—not available information; * number of molecules is indicated where greater than one.

**Table 5:** Summarized technical data and key findings from the reviewed MD simulations. The inclusion of years highlights the evolution of technical methods, computational resources, and the resulting research insights over the past twelve years.

FF/Water Model	Duration per System, ns	Aβ Length/PDB ID/Type (Monomer/Dimer/ (Proto-) Fibril)	Inhibitor *	Main Findings	Year, Ref.
OPLS-AA/L/TIP3P	(1) 5 (1117 replicas) (2) 15 (550 replicas) (3) (i) and (iii) 200, (ii) 180 (4) (j) 30, (jj) and (jjj) 100	Aβ_16-22_/STDR—simulated/ (1) monomer—1117 conformations (2) monomer—550 conformations (3) four dispersed monomers (tetramer); (4) manually constructed β-oligomer (16-mer)	*s*INO (1) in ratio 2:1 (2) in ratio 15:1 (3) in ratios (i) 1:2, (ii) 15:4, and (iii) 45:4 (4) in ratios (j) 4:16, and 64:16 at concentrations (jj) 15 mM and (jjj) 52 mM of the peptide	(1) and (2) bind weakly and reversibly, do not bind cooperatively; predominantly form nonpolar contacts with F; H-bonds with E (3) disordered oligomers are formed despite *s*INO ratio and concentrations, mostly in random coil conformation, with unaffected morphology and no effect on the aggregation kinetics (4) (j) binds predominantly at the faces of the β-oligomer with nonpolar groups of F and K and with the charged groups of K and E; does not penetrate the β-sheet core; at (jj) and (jjj) forms clusters that bind to oligomer	2013 [420]
CHARMM22 */SPC	100	Aβ_17-42_/2BEG/pentamer	CAF	destabilizes fibril structure; decreases β-sheet content, predominantly the β2 region; reduces H-bonds; interrupts the D23–K28 salt bridges, decreases inter-residual hydrophobic contacts	2019 [421]
Amber99-ILDN/TIP3P	150	Aβ_11-42_/2MXU/pentamer, fibril	SGL	intercalated between adjacent peptide chains, leading to fibril destabilization; reduces the β-structure content, fibril order, and inter-chain interaction surface; interacts with H14–G25 region	2020 [206]

As most acronyms are identical to those in the previous table, only the new ones are listed below: STDR—simulated tempering distributed replica sampling algorithm; *s*INO—*scyllo*-inositol, CAF—caffeine, and SGL—6-shogaol. * number of molecules is specified when different from 1.

**Table 6:** Key findings from MD simulations by scaffold group and Aβ form.

Scaffold	Cmpd	Monomeric	U-Shaped	S-Shaped	LS-Shaped
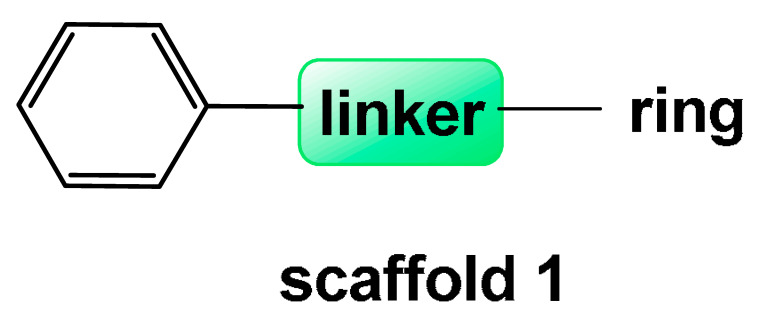	CCN	moves around monomeric Aβ; multiple contacts like H-bonds, π–π stacking, hydrophobic and cation–π interactions with numerous residues: F4–D7, Y10, E11, H13, H14, K16, L17, F19–A21, L24, K28, I31, G33–M35 [199,200,201,202,210]	destabilizes peripheral chains than central ones; disrupts the D23–K28 salt bridge; binds preferably to the C-terminal residues; specific interactions to F19 (π–π stacking) and H-bonds with E22 (from the loop), M35 (from β2), and G37 (from the C-terminal) [204]; reduces β-sheet; increases unorganized regions of the outermost chains; decreases number of intermolecular backbone H-bonds [205]; at higher concentration: individual or stacked CCN molecules deposit parallel- (predominant) or perpendicular-mode the fibril axis; enters the fibril hydrophobic core by the open ends or binds to the loop region between two β-strands [210].	breaks down all β-sheets within the ^12^ VHHQKLVFF ^20^ domain of the outermost peptide; partial dissociation of the fibril structure; [207]; inserts into a pocked within the fibril, disrupts the ordered fibril structure, decreases β-sheet content, fibril order and inter-chain interaction surface, binds to E11–F19 and I32–L24 areas [206]	
	Wgx-50		binds in the concave region of β-strand–loop–β-strand motif; disrupted the cross-β-sheet subunit, structural distortion between the first two peptide subunits; increased inter-peptide distances; promoted protofibril destabilization; a reduced number of interchain backbone H-bonds, increased hydrophobic contact distances between A21 and V36, and partial disruption of the D23–K28 salt bridge [212]		
	RESV	prevents monomer aggregation; significantly reduces β-sheet; H-bonds with Q15 and D23; contacts with V12, H13, Q15, K16, F19, F20, D23, V24, N27, A30, I31, and L34; alters the distance between M35 and I31, leading to destabilization of α-helical structure [213]	disrupts fibrillar structure, leading to unordered non-toxic aggregates; reduces the β-sheet; reduces α-helix and β-sheet content; H-bonds with F19, F20, A21, V36, G37, and G38; interacts with F19, F20, A21, L34, M35, V36, G37, G38, and A40; increases the distance between the S atom of M35 and the carbonyl O of I31; helical destabilization, reducing the likelihood of sulfuranyl radical formation [213]		
	PCT			disrupts the fibril by inserting into a pocket formed by the S-shaped fibril; induces conformational disorder of the entire fibril structure; reduces β-sheet content and inter-chain interaction surfaces; binds to E11–F19 and I32–L24 areas [206]	
	OLEU			destabilizes the whole fibril as it intercalates between adjacent peptide chains; reduces β-sheet content and inter-chain interaction surfaces; contacts V18–V24 and N27–I31 [206]	
	OleuA		strongly destabilizes and perturbs preformed fibrils, complete fibril disassembly; disrupts the D23–K28 salt bridge; increases interchain distances and unstructured peptide conformations; reduces the number of inter-backbone H-bonds and significantly the β-sheet content; primarily interacts with residues L17–D23, K28, and I31–L34 [214]		
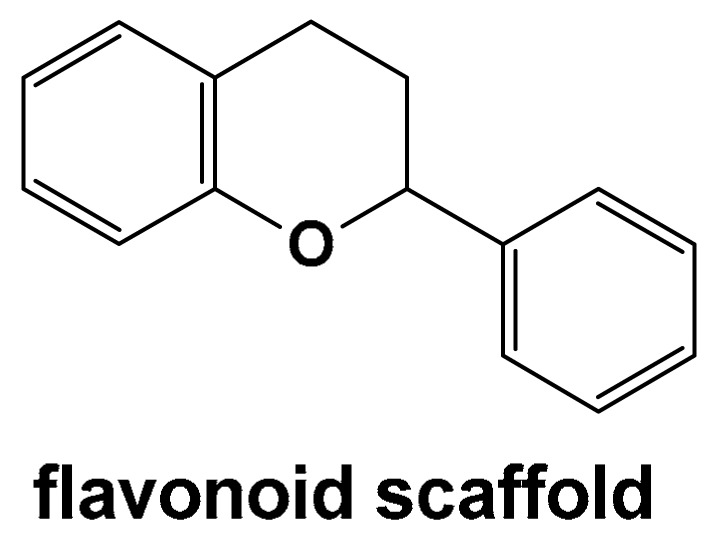	EGCG	inhibits β-sheet dose-dependently; key contact residues: F4, R5, F19, F20, K28, G29, L34, M35, V36, G37, and I41; H- bonds with F20, K28, G29, G37, and I41; hydrophobic contacts with F4, F20, K28, G29, M35, and I41 [302]			located on the surface; disrupts N-terminal (D1–G9) and C-terminal (K28–A42) segments; increases the kink angle around Y10; decreases H-bonds in the H6–E11 segment, increasing them in the E11–H13; interrupts the stabilizing K28–A42 salt bridge; interacts with F4, R5, D7, Y10, E11, H13, H14, K28, L34, I41, and A42; key interactions: H-bonds with E11, π–π stacking with H14 and Y10, with the COO^−^ group of A42 and a cation–π contact with K28 [308]; 4 BSs defined by residues: F19 for BS1 (inside the fibril), E3—for BS2 (near the N-terminus), I41—BS3 (near the C-terminus), and E11 and H13 for BS4 (within the fibril) [310]
	MYR	destabilizes the U-shaped monomer; H-bonding is dominant; changes β-sheet content into coil; penetrates Aβ core; interacts with CO and NH groups; forms self-clustered Aβ–MYR complexes at higher molar ratios [30,311]	remodels rigid, toxic protofibrils into expanded, fragile, and nontoxic amorphous aggregates by reducing the number of interchain hydrogen bonds; the β-sheets remained intact, but the rigid “steric-zipper” motif was significantly disrupted [30]		
	MOR	destabilizes the U-shaped monomer; H-bonding is dominant; changes β-sheet content into bends or coils; penetrates Aβ core; interacts with CO and NH groups; forms self-clustered Aβ–MOR complexes at higher molar ratios [30,311]; minimal impact on the monomeric structure at low molar ratio; at the higher molar ratio: significantly alters both the secondary and tertiary structures; covers Aβ_1-42_, restricting it from collapse and α-helix and β-sheet interconversion; altered structures may represent the early formation of “off-pathway” aggregates [297]	remodels rigid, toxic protofibrils into expanded, fragile, and nontoxic amorphous aggregates by reducing the number of interchain hydrogen bonds; the β-sheets remained intact, but the rigid “steric-zipper” motif was significantly disrupted [30]		
	GEN		stabilizes protofibril; reduces β-sheet content; contacts L17, F20, E22, I31, G33, M35, and V39; preferably locates at the C-terminal β-sheet groove near G33 and M35 [314]		
	GOS			inserts into a pocket within the fibril, disrupts the ordered fibril structure, decreases β-sheet content, fibril order and inter-chain interaction surface, binds to E11–F19 and I32–L24 areas [206]	
	biflavonouds			induces conformational changes in the fibril, leading to disaggregation; form π–π interactions with F4, H6, Y10, H13, and H14; significant reduction in β-sheet content with a key OH group at the R2 and R3 positions involved in H-bonds with the peptide backbone [315]	
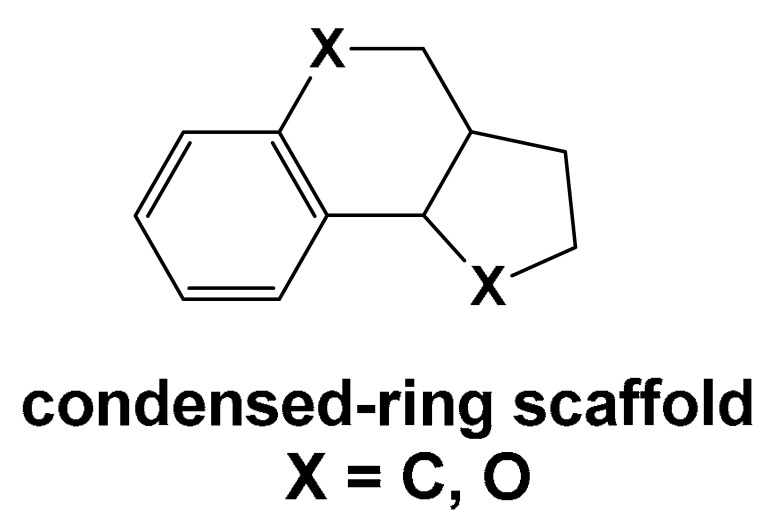	TS1	binds to DR; decreases β-sheet content, contacts with F4, D7, Y10, K16, F20, K28, and V40 [407]	inhibit Aβ aggregation through multiple mechanisms; two BSs: A1—at the β-sheet groove at on the external surface of the hydrophobic C-terminal β-sheet (I31–M35); A2—near the N-terminus (F4–H6); forms contacts with: F4, H6, I31, G33, L34, M35; [406]; inhibits fibril growth as disrupts β-sheet integrity; reduces fibril stability; hinders lateral association of Aβ aggregates; mainly locates in DR; increases twist angle; disappears the bend region (^37^ GGV ^39^) [407]		
	TS2		inhibit Aβ aggregation through multiple mechanisms; seven BSs (B1–B7), BS1(≡A1 for TS1) and BS6 (≡A2 for TS2), cover ~ 30% of pose population, forms contacts with F4, H6, Y10, V39, F40, I41 [406]		
	brazilin		fibril destabilization and remodelling, preventing further fibril formation; multiple BSs; preferentially interacting with residues L17, F19, F20, and K28; strong hydrophobic interactions between the phenyl rings of brazilin and F20; OH groups formed over ten H-bonds; disrupts the D23–K28 salt bridge as forms H-bond with K28; reduces the interchain backbone H-bonds [31]		

## Data Availability

No new data were created or analyzed in this study. Data sharing is not applicable to this article.

## Abbreviations

AD	Alzheimer’s disease
AFM	Atomic force microscopy
aMD	accelerated molecular dynamics
AMF	amentoflavone
APP	amyloid-β (A4) precursor protein
ATP	adenosine triphosphate
BACE-1	β-amyloid precursor (APP)-cleaving enzyme 1
BBB	blood–brain barrier
BDNF	brain-derived neurotrophic factor
BIL	bilobetin
BS	binding site
CAF	caffeine
CCN	curcumin
CD	Circular dichroism
CG	coarse-grained
CGM	coarse-grained model
cMD	classical/conventional molecular dynamics
CNS	central nervous system
CTF	C-terminal domain of APP
DMD	discontinuous molecular dynamics
DR	disordered region
EGCG	(-)-epigallocatechin-3-gallate
FAD	familial Alzheimer’s disease
FF	force field
GEN	genistein
GOS	gossypin
gx-50	wgx-50, lemairamin, N-[2-(3,4-dimethoxyphenyl)ethyl]-3-phenylacrylamide
IDP	intrinsically disordered proteins
INO	inositol
MD	molecular dynamics
MOR	morin
MYR	myricetin
NMR	nuclear magnetic resonance
OLEU	oleuropein
OleuA	oleuropein aglycone
PCF	podocarpusflavone
QUER	quercetin
RA	rosmarinic acid
REM	replica exchange method
REMD	replica exchange molecular dynamics
REST	replica exchange solute tempering
RESV	resveratrol
REXAMD	replica exchanged accelerated molecular dynamics
RONS	reactive oxygen and nitrogen species
ROS	reactive oxygen species
RPMD	replica permutation molecular dynamics
SAD	sporadic Alzheimer’s disease
SGL	6-shogaol
*s*INO	*scyllo*-inositol
SQF	sequoiaflavone
SPC	simple point charge water model
SPCE	extended simple pint charge water model
STF	sotetsuflavone
STDR	simulated tempering distributed replica
TIP3P	transferable intermolecular potential 3-point water model
TS0, TS1, TS2	tanshinones 0, 1, and 2
wgx-50	gx-50, lemairamin, N-[2-(3,4-dimethoxyphenyl)ethyl]-3-phenylacrylamide
